# Targeted extracellular degradation of LRP8 promotes ferroptosis in cancer cells

**DOI:** 10.1073/pnas.2616933123

**Published:** 2026-09-30

**Authors:** Fangzhu Zhao, Alex Inague, Trenton M. Peters-Clarke, Yifei Chen, Snehal D. Ganjave, Yun Zhang, Kun Miao, Zi Yao, Yan Wu, Madison K. C. Seto, Kevin K. Leung, James A. Olzmann, James A. Wells

**Affiliations:** ^a^ Department of Pharmaceutical Chemistry, University of California San Francisco, San Francisco, CA 94158; ^b^ Department of Molecular and Cell Biology, University of California Berkeley, Berkeley, CA 94720; ^c^ Department of Metabolic Biology and Nutrition, University of California Berkeley, Berkeley, CA 94720; ^d^ Department of Cellular and Molecular Pharmacology, University of California San Francisco, San Francisco, CA 94158

**Keywords:** targeted protein degradation, antibody engineering, LRP8, ferroptosis

## Abstract

Tumor reliance on antioxidant defenses creates a vulnerability to ferroptosis, yet strategies to therapeutically disable these systems remain limited. Here, we identify targeted degradation of the selenium uptake receptor lipoprotein receptor-related protein 8 (LRP8) as an effective approach to decrease the abundance of the ferroptosis-protective enzyme glutathione peroxidase 4 (GPX4). Using bispecific cytokine receptor–targeting chimeras (KineTACs) that couple LRP8 to cytokine receptor internalization pathways, we selectively direct LRP8 to the lysosome for degradation. LRP8 degradation reduces the abundance of several selenoproteins, including GPX4, lowering the cellular threshold for lipid peroxidation and sensitizing cancer cells to ferroptosis. These findings establish receptor-mediated selenium uptake as a critical, targetable node in ferroptosis resistance and demonstrate that extracellular protein degradation can be leveraged to reprogram intracellular translational dependencies in cancer cells. More broadly, this work provides a framework for exploiting nutrient acquisition pathways to overcome therapy resistance.

## Significance

Many therapy-resistant cancers depend on the selenium-containing enzyme glutathione peroxidase 4 (GPX4) to suppress ferroptosis, an iron-dependent form of cell death driven by lipid peroxidation. Direct inhibition of GPX4 has proven challenging because of toxicity and limited drug-like properties of existing inhibitors. Here, we show that targeted degradation of the selenium uptake receptor lipoprotein receptor-related protein 8 (LRP8) reduces intracellular GPX4 abundance by disrupting selenium acquisition, thereby sensitizing cancer cells to ferroptosis. We demonstrate that extracellular targeted protein degradation (eTPD) of a cell surface receptor can indirectly reprogram intracellular metabolic and translational pathways. These findings establish the selenoprotein P–LRP8 axis as a therapeutic vulnerability and broaden the scope of eTPD as a strategy for modulating intracellular protein networks through extracellular targets.

Ferroptosis, an iron-dependent form of regulated cell death characterized by the catastrophic accumulation of oxidatively damaged lipids (i.e., lipid peroxides), has emerged as a promising therapeutic target for treatment-resistant cancers (1–3). Several tumor types exhibit heightened sensitivity to ferroptosis induction, such as clear cell renal cell carcinoma (4), MYCN-amplified neuroblastoma (5–8), drug-tolerant persister cells (9–11), and metastasizing cancer cells (12). Ferroptosis induction not only eliminates treatment-refractory cancer populations but may also synergize with existing modalities, such as radiotherapy, immunotherapy, and targeted inhibitors.

Many cancers possess defense systems that promote ferroptosis resistance. The primary mechanism is mediated by the glutathione peroxidase 4 (GPX4) (13, 14), which directly reduces lipid hydroperoxides to inert lipid alcohols. Complementary radical-trapping antioxidant systems, such as the ferroptosis suppressor protein 1 (FSP1)–coenzyme Q10 (CoQ10) pathway (15, 16) and the GTP cyclohydrolase 1 (GCH1)–tetrahydrobiopterin (BH4) axis (17, 18), further act to prevent the propagation of lipid peroxidation. The catalytic activity of GPX4 depends on a selenocysteine residue in its active site, which is incorporated via a specialized translation process that recodes UGA stop codons as selenocysteine insertion sites (19, 20). Adequate selenium supply is therefore essential for sufficient GPX4 expression and activity. Selenium is delivered either as inorganic selenite or through selenoprotein P (21) (SEPP1, also known as SELENOP, which naturally contains 10 selenocysteines) endocytic uptake mediated via its receptors, such as the low-density lipoprotein receptor-related protein 8 (LRP8), and subsequent lysosomal breakdown (19, 22). Recent work indicates that LRP8 is important for tumor growth and ferroptosis resistance, including MYCN-amplified neuroblastoma (22, 23) and triple-negative breast cancer (TNBC) (22–24), acting to provide rapidly growing cancer cells with sufficient selenium pools for GPX4 translation. Loss of LRP8 results in ribosome stalling at the selenocysteine codon of GPX4, ultimately leading to ribosome collisions, premature translation termination, and reduced GPX4 abundance (24). Functionally, the reduction in GPX4 translation sensitizes cells to ferroptosis. Remarkably, in MYCN-amplified neuroblastoma, LRP8 loss is sufficient to trigger ferroptosis both in culture and in orthotopic xenograft models (23), underscoring its potential as a therapeutic target.

Recently advances in extracellular targeted protein degradation (eTPD) have exploited natural endolysosomal and/or proteasomal pathways to eliminate both membrane-bound and soluble proteins (25–30). These bifunctional antibodies harness diverse cell surface recycling and degradation mechanisms to selectively remove extracellular targets, thereby expanding the druggable proteome to include plasma membrane proteins essential for tumor survival. Building on this concept, we developed bispecific antibody degraders that induce the selective degradation of the SEPP1 receptor, LRP8. By disrupting the SEPP1–LRP8 axis using the cytokine receptor-targeting chimeras (KineTACs) (27), these degraders reduce GPX4 levels, thereby sensitizing cancer cells to ferroptosis. This strategy establishes a tractable framework for therapeutic targeting of ferroptosis resistance and highlights the potential of antibody-based protein degradation to capitalize upon key vulnerabilities in cancer.

## Results

### Engineering Antibodies Against LRP8.

**Fig. 1.**
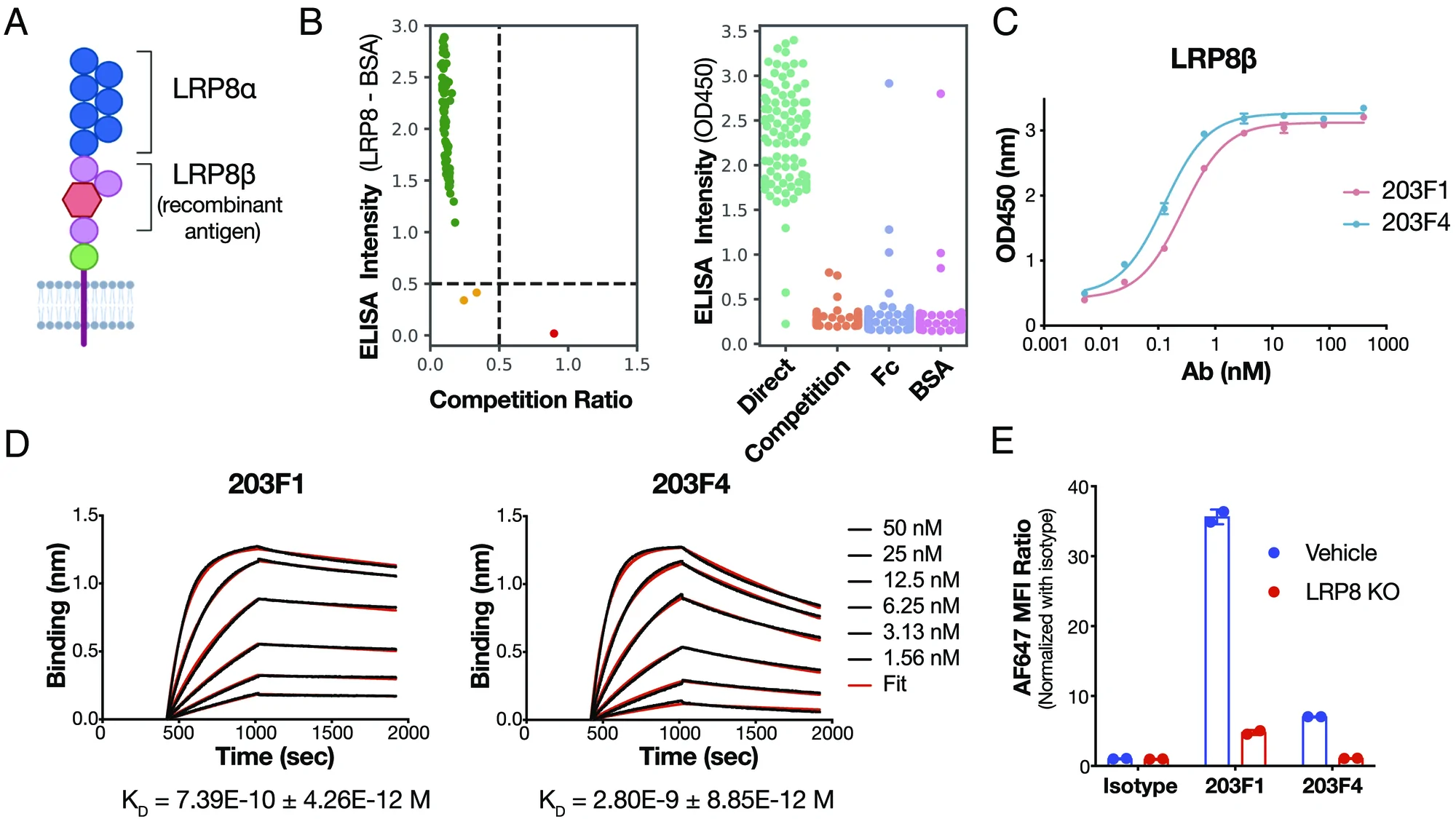
Generation and characterization of LRP8-specific antibodies. (*A*) The construct of full-length human LRP8, and the LRP8β was expressed as recombinant antigen with TEV-Fc-Avitag fusion for antibody selection by phage display. (*B*) Screening hits from phage selection. Plot on left shows screening phage for those that express well and are blocked from binding to antigen coated plate by soluble antigen in solution (upper left quadrant). Plot on right shows results from further testing in separate enzyme-linked immunosorbent assay (ELISA) by direct antigen binding, competition with soluble antigen, direct binding to Fc, or bovine serum albumin (BSA). (*C*) ELISA binding of purified recombinant selected Fabs against the LRP8 antigen. (*D*) Biolayer interferometry (BLI) analysis of 203F1 and 203F4 Fabs against the recombinant LRP8β antigen. Biotinylated LRP8β was immobilized via the streptavidin biosensor and varying concentrations of each Fab were injected. Black lines are the experimental trace obtained from the BLI experiments and colored lines are the global fits. (*E*) Flow cytometry analysis of LRP8 IgG binding on HCC1143 control cells or LRP8 knockout (KO) cells. Cells were stained with LRP8 antibodies for 30 min, followed by binding AF647 conjugated anti-human IgG (H+L) antibody staining for 15 min. The ratio of mean fluorescent intensity (MFI) of the APC fluorescence channel was normalized to isotype control antibody. Data represent mean ± SD from two biological replicates.

It has been reported that SEPP1 binds to the β-propeller domain of LRP8 (31). To target this region, we selected antibodies against the epidermal growth factor (EGF)-like domain of LRP8 (LRP8β), which encompasses the β-propeller domain (Fig. 1*A*). We expressed biotinylated LRP8β using a tobacco etch virus (TEV)-Fc-Avitag construct to enable phage clearance via TEV protease cleavage (32). Phage displayed fragment antigen-binding antibody (Fab) libraries were subjected to iterative rounds of selection against immobilized LRP8 antigen, with preclearing steps to remove nonspecific and Fc binding clones. Enriched binders were recovered after each round and amplified under increasingly stringent conditions to isolate high affinity, antigen-specific antibodies (32).

After four rounds of phage selection, we plated 96 single phage colonies for screening using an ELISA (32). Clones that specifically bound recombinant LRP8β-Fc but not Fc, BSA, or soluble LRP8β in competition assays (Fig. 1*B*) were sequenced and reformatted as recombinant Fabs. We identified four unique anti-LRP8 antibody clones, all of which bound MDA-MB-231 cells with endogenous LRP8 expression by flow cytometry and recombinant LRP8 by ELISA (*SI Appendix*, Fig. S1 *A*–*D*). None of the Fabs exhibited cross-reactivity with low-density lipoprotein receptor (LDLR), LRP2 or the N-terminal ligand binding domain of LRP8 (LRP8α) (*SI Appendix*, Fig. S1 *E*–*G*). All Fabs exhibited minimal polyreactivity against nonspecific antigen panels (33) (*SI Appendix*, Fig. S2*A*), and were monodispersed with analytical size exclusion chromatography (*SI Appendix*, Fig. S2*B*). Given that 203F1 and 203F4 clones displayed high affinities against LRP8β, we characterized them in further detail. ELISA assays also confirmed strong binding of both clones to LRP8β (Fig. 1*C*), with EC_50_ values of 0.19 nM and 0.28 nM, respectively. BLI measurements demonstrated that 203F1 and 203F4 bound LRP8 with affinities of 0.74 nM and 2.8 nM, respectively (Fig. 1*D*). Epitope binning using BLI revealed that 203F1 and 203F4 could bind LRP8β simultaneously, indicating they recognize distinct, noncompeting epitopes (*SI Appendix*, Fig. S3*A*). Both antibodies selectively bound to LRP8-positive HCC1143 cells but not to LRP8 knockout cells, further supporting their specificity (Fig. 1*E* and *SI Appendix*, Fig. S3 *B* and *C*).

### LRP8 Antibody Blocks the Uptake of Selenoprotein P.

**Fig. 2.**
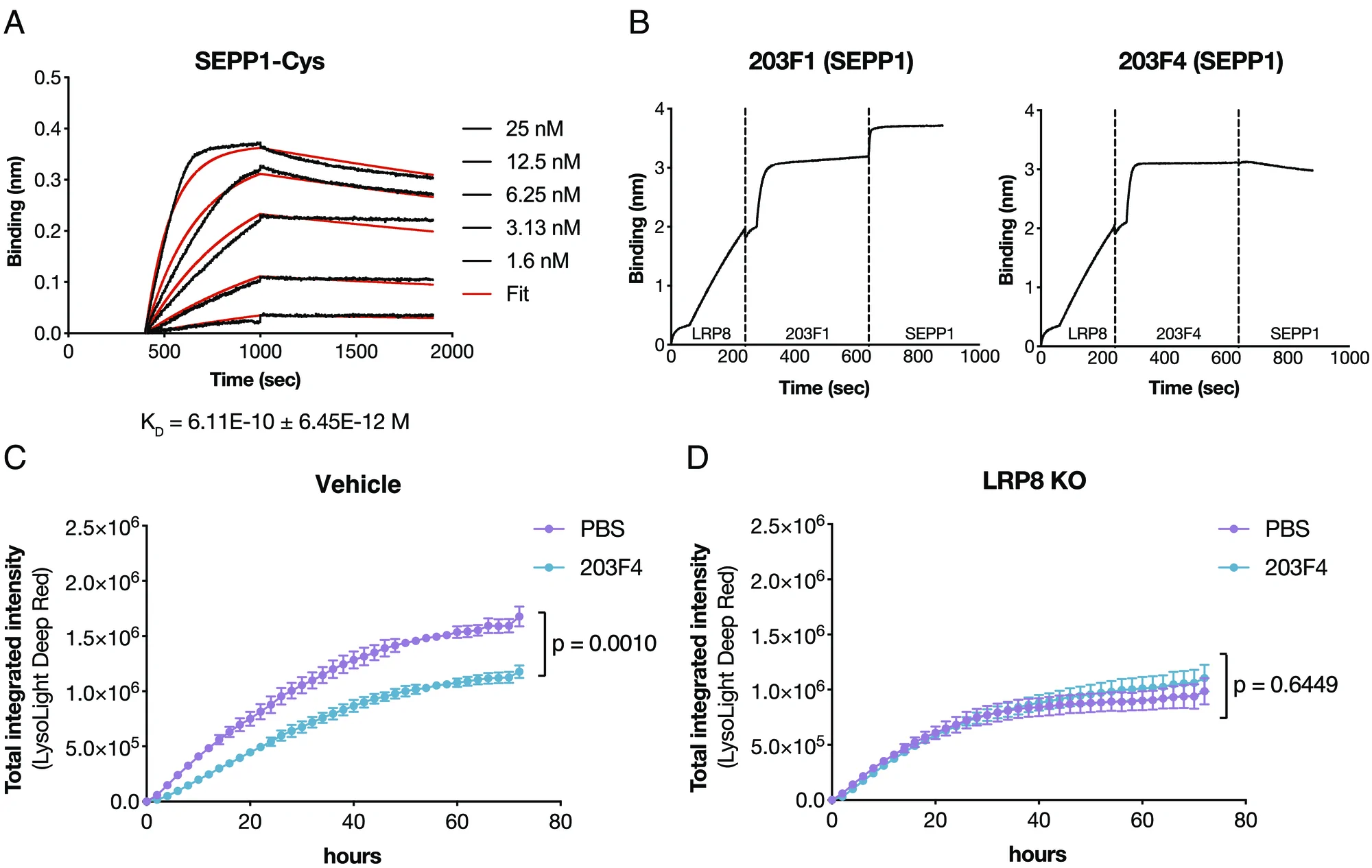
The 203F4 clone binds specifically to cells and blocks selenoprotein P binding. (*A*) BLI analysis of SEPP1-Cys against the recombinant LRP8β antigen. Biotinylated LRP8β was immobilized via the streptavidin biosensor and varying concentrations of SEPP1-Cys were injected. Black lines were the experimental trace obtained from the BLI experiments and colored lines were the global fits. (*B*) Epitope binning of SEPP1-Cys and LRP8 Fabs revealed two different epitopes on LRP8β. Biotinylated LRP8β was captured using a streptavidin biosensor and indicated Fabs at a concentration of 200 nM were incubated for 10 min followed by incubation with 50 nM of the competing SEPP1-Cys for 5 min. (*C* and *D*) Internalization of SEPP1-Cys in HCC1143 cells with control sgRNA (vehicle) or LRP8 sgRNA (knockout). SEPP1-Cys was labeled with the lysolight deep red dye (LLDR) to monitor lysosomal trafficking. Cells were preincubated with phosphate-buffered saline (PBS) or 200 nM 203F4 Fab for 30 min prior to 50 nM SEPP1-Cys-LLDR treatment. Images were captured every 2 h for 72 h on the Incucyte. Total integrated intensity was calculated by NIRCU × μm^2^/image. Data represent mean ± SD from four biological replicates. Statistics were calculated by one-way ANOVA and Holm–Sidak multiple comparison tests.

To assess whether LRP8 antibodies interfere with SEPP1 binding, we expressed a recombinant cysteine-substituted variant of selenoprotein P (SEPP1-Cys), in which all 10 selenocysteine (Sec) residues encoded by the UGA stop codon were replaced with cysteines to facilitate expression (21). BLI showed that SEPP1-Cys bound LRP8β with a high affinity of 0.61 nM (Fig. 2*A*). AlphaFold3 modeling of the SEPP1–LRP8 complex yielded a high-confidence interface (ipTM = 0.80), consistent with a stable interaction (*SI Appendix*, Fig. S4*A*). Top-ranked models consistently predicted engagement of both SEPP1 domains with the LRP8 β-propeller, supported by Rosetta interface energy scoring (dG_separated = −53 REU) (*SI Appendix*, Fig. S4*A*). Epitope binning revealed that 203F4 clone competed with SEPP1-Cys for binding whereas 203F1 did not (Fig. 2*B* and *SI Appendix*, Fig. S4*B*).

We then used an Incucyte-based assay to monitor the uptake of SEPP1. We conjugated SEPP1-Cys with the cathepsin-dependent fluorescent probe, LLDR (34), which generates fluorescent signals upon cleavage by lysosomal cathepsins. We serum starved the HCC1143 cells, pretreated with PBS or LRP8 antibodies, then added SEPP1-LLDR. Importantly, the 203F4 antibody significantly reduced SEPP1 uptake (Fig. 2*C*). The blocking activity is abolished when 203F4 antibody was added to LRP8 KO cells, further indicating its binding dependency on LRP8 (Fig. 2*D*). We reasoned that the residual internalization of SEPP1 may occur via macropinocytosis. To test this, we treated cells with cytochalasin D (35), an actin polymerization inhibitor that suppresses membrane ruffling and macropinocytosis. Cytochalasin D almost completely abolished SEPP1 uptake, supporting the existence of a secondary macropinocytic pathway (*SI Appendix*, Fig. S4*C*). In contrast, the 203F1 antibody had no effect on SEPP1 internalization, consistent with its recognition of a distinct, noncompetitive epitope (*SI Appendix*, Fig. S4*D*).

### KineTACs Efficiently Degrade LRP8.

**Fig. 3.**
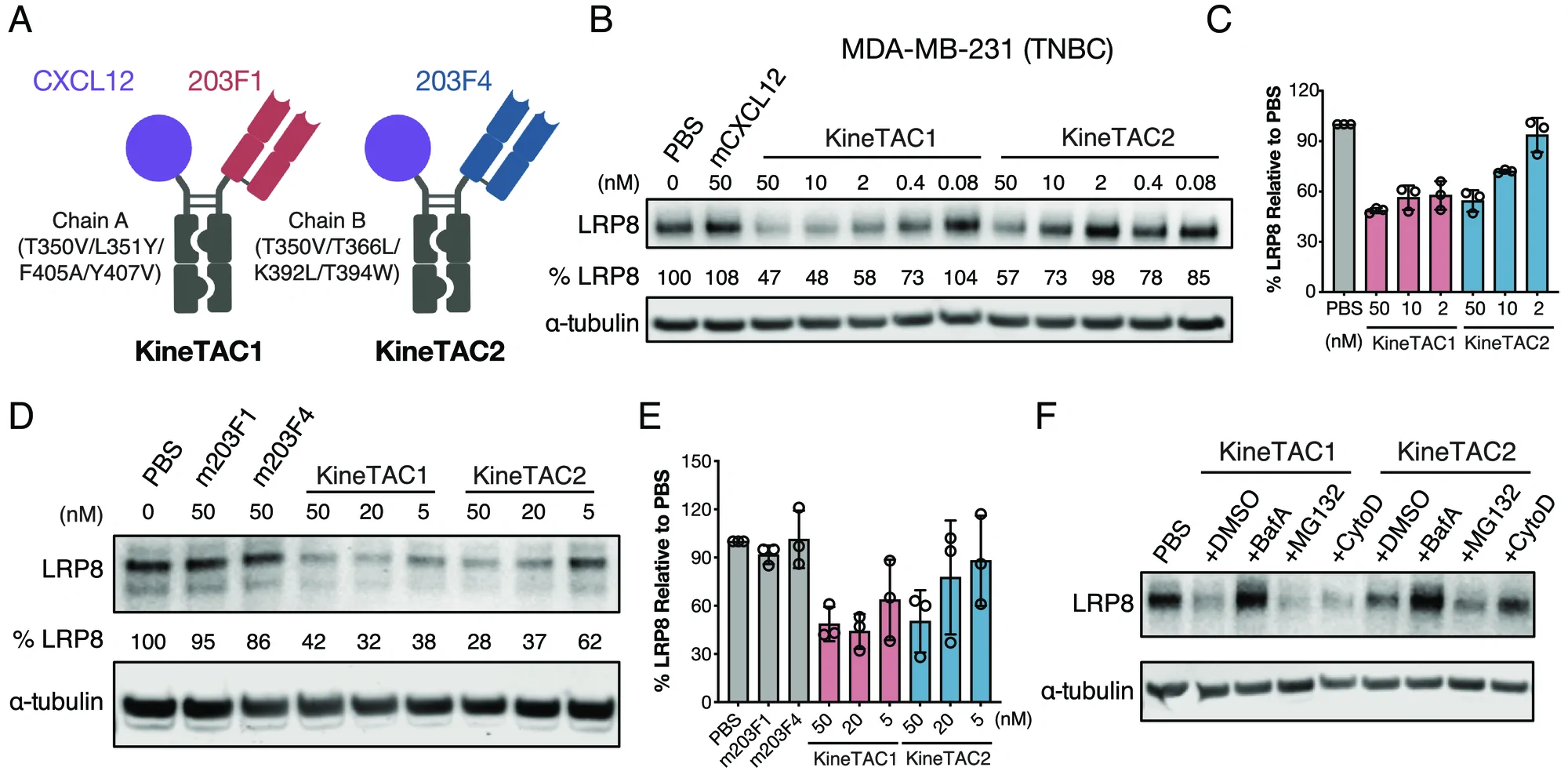
Generation of LRP8-degrading KineTACs. (*A*) Schematic illustration of bispecific LRP8-targeting KineTACs. Both Fc chains contain LALAPG mutations to inactivate effector functions. (*B* and *C*) Western blotting showing degradation of LRP8 in triple negative breast cancer MDA-MB-231 cells following various concentrations of KineTAC treatment for 24 h. Percent LRP8 levels were quantified by ImageJ relative to the PBS control. Data represent mean ± SD from three biological replicates. (*D* and *E*) Western blotting showing degradation of LRP8 in neuroblastoma SK-N-DZ cells following various concentrations of KineTAC or monomeric antibody treatment for 24 h. Percent LRP8 levels were quantified by ImageJ relative to the PBS control. Data represent mean ± SD from three biological replicates. (*F*) Western blot analysis showing lysosome-dependent LRP8 degradation on HCC1143 cells following KineTAC treatment. Cells were pretreated with either 500 nM Bafilomycin A (BafA), 500 nM MG132, or 500 nM cytochalasin D (CytoD) for 1 h followed by 24 h treatment with 50 nM KineTAC1 or KineTAC2.

To selectively target LRP8 for degradation, we generated two bispecific LRP8-targeting KineTACs (27), with one CXCL12 arm recruiting the decoy chemokine receptor CXCR7 (36, 37) or the signaling chemokine receptor CXCR4 (38), and the other anti-LRP8 arm of 203F1 (termed KineTAC1) or 203F4 (termed KineTAC2), respectively (Fig. 3*A*). To avoid Fc effector functions, LALAPG mutations were introduced in both Fc chains (39).

LRP8 is highly expressed in TNBC compared to other breast cancer subtypes and its genomic locus is amplified in 24% of TNBC tumors (40). In TNBC MDA-MB-231 cells, KineTAC1 induced robust LRP8 degradation within 24 h, with activity detectable at concentrations as low as 2 nM (Fig. 3 *B* and *C*). By contrast, KineTAC2 was markedly less potent, requiring ~50 nM for measurable degradation. The reduced activity likely reflects both weaker binding affinity of 203F4 and differences in epitope recognition. Similar results were obtained in HCC1143 cells and neuroblastoma SK-N-DZ cells, where KineTAC1 efficiently degraded LRP8 while KineTAC2 displayed reduced potency (Fig. 3 *D* and *E* and *SI Appendix*, Fig. S5 *A* and *B*). Monovalent LRP8 antibodies had no effect, indicating that degradation requires the CXCL12 arm for induced proximity. Given that LRP8 is an endocytic LDLR-family receptor, it may recycle to the plasma membrane upon antibody binding rather than undergoing self-degradation (41).

To further define the degradation mechanism, HCC1143 cells were pretreated with different pathway inhibitors prior to KineTAC exposure. LRP8 degradation was blocked by the lysosomal V-ATPase inhibitor, bafilomycin A1 (42), but not by the proteasome inhibitor MG132 (43) or the macropinocytosis inhibitor cytochalasin D (Fig. 3*F* and *SI Appendix*, Fig. S5*C*). These findings confirm that KineTAC-mediated LRP8 degradation proceeds through a lysosome-dependent pathway, consistent with prior reports of extracellular protein degradation (27, 41).

### LRP8 Degradation Reduces Selenoprotein Levels.

**Fig. 4.**
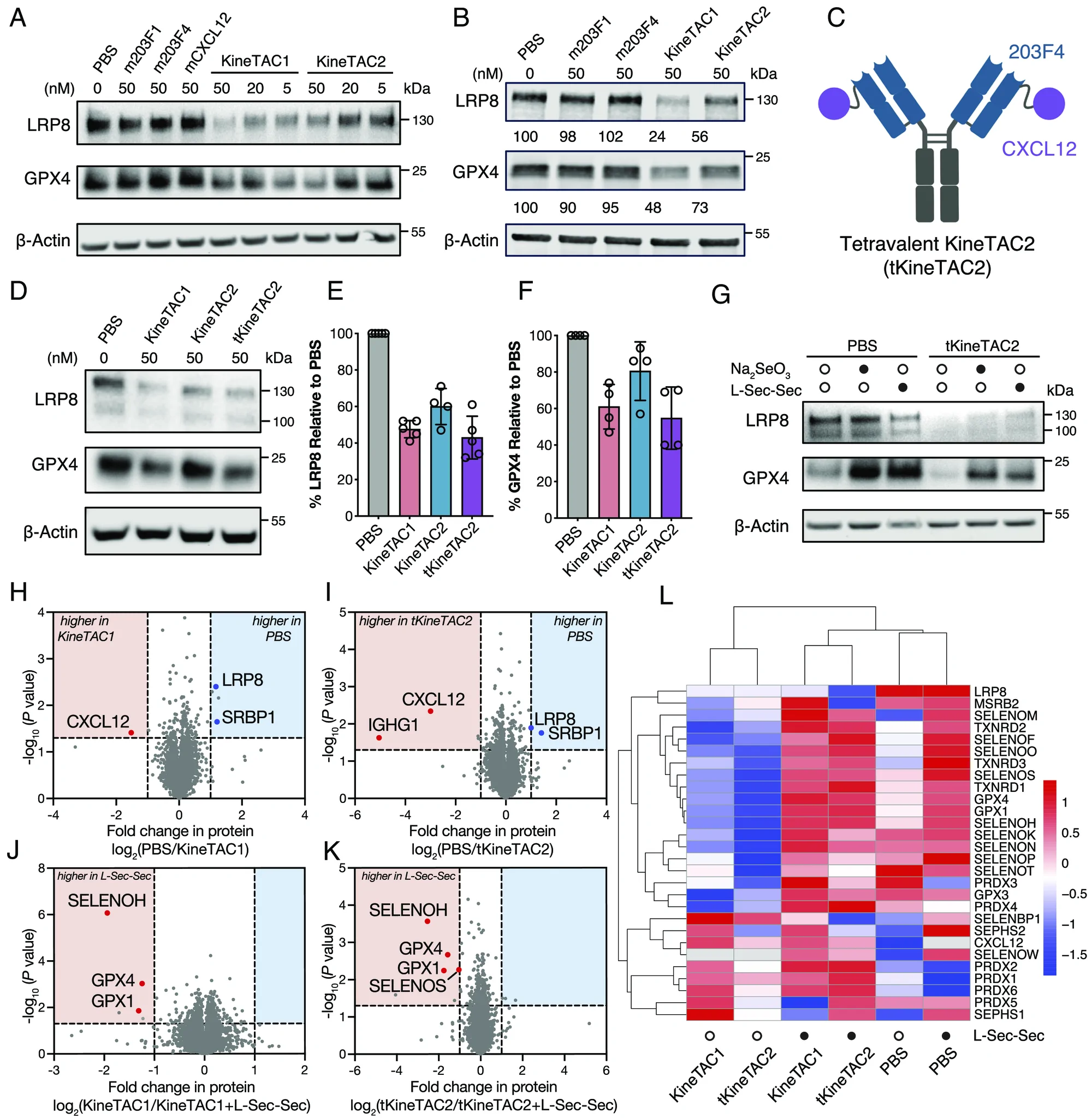
LRP8 degradation reduces GPX4 and selenoprotein translation. (*A*) Western blotting shows that KineTACs degrade LRP8 causing reduction in GPX4 levels in HCC1143 cells after 48 h of KineTAC treatment, whereas treatment with monomeric antibodies have no such effect. Data are representative for at least three independent experiments. (*B*) Western blotting showing degradation of LRP8 and reduction of GPX4 in HT-1080 cells following 50 nM KineTACs treatment for 72 h but not for monomeric antibodies (m203F1, m203F4). (*C*) Schematic of the tetravalent KineTAC2 (tKineTAC2). (*D*–*F*) Western blotting showing degradation of LRP8 and reduction of GPX4 in Kelly cells (*D*) following 50 nM KineTAC treatment for 72 h. Quantification of LRP8 (*E*) and GPX4 (*F*) protein levels relative to a PBS control was performed using ImageJ. Data represent mean ± SD from four biological replicates. (*G*) Western blotting showing that selenium supplementation restores selenoprotein abundance following 72 h of 50 nM tKineTAC2 treatment. GPX4 levels could be rescued by addition 50 nM sodium selenite and 50 nM L-selenocystine (L-Sec-Sec) despite ongoing LRP8 degradation. (*H*–*K*) Whole-cell proteomics of Kelly cells treated for 48 h with PBS, 50 nM KineTAC1 (*H*), 50 nM tKineTAC2 (*I*), or the same treatments with 50 nM L-Sec-Sec supplementation (*J* and *K*). Fold changes in protein levels were quantified by mass spectrometry. *P* values were calculated using two-tailed Student’s *t* tests. (*L*) Heatmap showing abundance of selenocysteine-containing and related proteins in Kelly cells after 48 h of treatment with PBS, KineTAC1, or tKineTAC2, with or without 50 nM L-Sec-Sec (filled dots and open dots, respectively).

To determine whether LRP8 degradation affects selenoprotein translation, we treated HCC1143 cells with KineTAC1, KineTAC2, or the single armed monomeric antibodies for 48 h. KineTAC1 efficiently degraded LRP8, whereas KineTAC2 was less potent and only effective at 50 nM (Fig. 4*A*). Both treatments reduced GPX4 abundance, although KineTAC2 was less effective, suggesting that active degradation of LRP8 has a stronger impact on selenoprotein translation than merely blocking ligand binding. Similar results were observed in HT-1080 fibrosarcoma cells, where KineTAC1 markedly reduced LRP8 and lowered GPX4 by ~52% after 72 h, while KineTAC2 again showed weaker effects (Fig. 4*B*). Supplementation with sodium selenite or L-selenocystine restored GPX4 levels regardless of LRP8 degradation, indicating that reduced selenoprotein level arises from impaired selenium uptake (*SI Appendix*, Fig. S6 *A* and *B*).

Because the antibody 203F4 blocks SEPP1 uptake but lacks strong degradation activity, we sought to enhance avidity by fusing CXCL12 to the N terminus of its light chain, generating a tetravalent KineTAC2 IgG (termed tKineTAC2) (Fig. 4*C* and *SI Appendix*, Fig. S6*C*). This engineered antibody robustly degraded LRP8 and decreased GPX4 levels in neuroblastoma Kelly cells, achieving greater degradation than KineTAC1 (Fig. 4 *D*–*F*), indicating that improved avidity helps to increase degradation activity (44). Consistently, inorganic selenium supplementation restored selenoprotein levels regardless of tKineTAC2-mediated LRP8 degradation (Fig. 4*G* and *SI Appendix*, Fig. S6*D*).

To assess broader consequences of LRP8 degradation, we performed whole-cell proteomics in Kelly cells, given that MYCN-amplified neuroblastoma is particularly sensitive to LRP8-dependent selenium uptake. After 48 h of KineTAC treatment, LRP8 was the most significantly downregulated protein following KineTAC1 and tKineTAC2 treatment, underscoring the selectivity of KineTAC-mediated extracellular protein degradation (27) (Fig. 4 *H* and *I* and *SI Appendix*, Fig. S7). Interestingly, sterol regulatory element-binding protein 1 (SREBP1) (45, 46), a transcriptional factor that regulates lipid homeostasis, was also downregulated upon KineTAC treatment. It has been shown that LRP8 expression level is associated with lipid metabolism‐related genes, including SREBP1 (47). Consistent with western blot results, proteomics showed that L-selenocystine supplementation during KineTAC treatment rescued GPX4, GPX1, and other selenoproteins (Fig. 4 *J* and *K*), confirming their dependence on extracellular selenium. Overall, we observed more downregulation of a panel of selenoproteins including the SELENO family, the thioredoxin reductase (TrxR) family, and the GPX family (Fig. 4*L*). Interestingly, the peroxiredoxin protein (PRDX) abundance, such as PRDX1 and PRDX2 did not change; these proteins contain cysteines instead of selenocysteines as antioxidant enzymes (48), further indicating the dependence of Sec in the LRP8–GPX4 axis (Fig. 4*L*).

Together, these findings show that LRP8 degradation selectively impairs selenium metabolism, resulting in reduced GPX4 and other selenoproteins, while also inducing broader metabolic alterations in cells.

### LRP8 Degradation Sensitizes Tumor Cells to Ferroptosis.

**Fig. 5.**
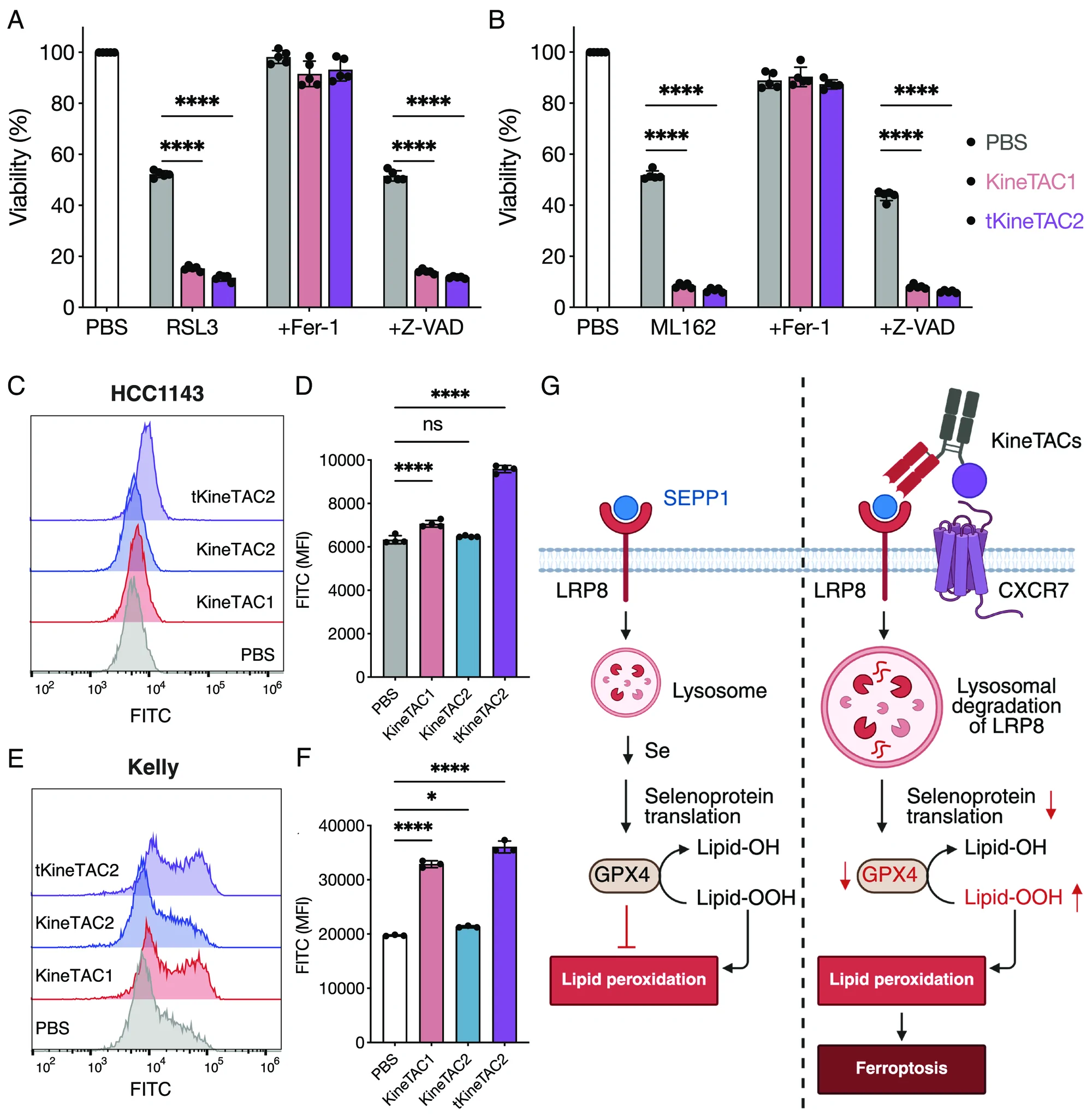
LRP8 degradation sensitizes tumor cells to ferroptosis. (*A* and *B*) Viability of HCC1143 cells treated with KineTACs for 24 h, followed by treatment with (*A*) 200 nM RSL3 or (*B*) 200 nM ML162 for additional 24 h, in the presence or absence of 1 μM Fer-1 or 5 μM Z-VAD-FMK. Cell viability was measured using the CellTiter-Glo Reagent. Data represent five technical replicates ± SD and are representative of at least three independent experiments. (*C* and *D*) Lipid peroxidation in HCC1143 cells pretreated with KineTACs for 72 h and then with 200 nM RSL3 for 5 h at 37 °C, detected using 5 μM BODIPY 581/591 C11 dye by flow cytometry. Gating was performed on singlets/live cells (*C*), and GFP MFI was quantified (*D*). Data represent quadruplicates ± SD. (*E* and *F*) Flow cytometry analysis of lipid peroxidation in Kelly cells pretreated with KineTACs for 72 h and then with 100 nM RSL3 for 5 h, detected using 5 μM BODIPY 581/591 C11 for 10 min at 37 °C, and counterstained with Near IR live/dead dye. Gating was performed on singlets/live cells (*E*), and GFP MFI was quantified (*F*). Data represent triplicates ± SD. (*G*) Schematic showing that lysosomal degradation of LRP8 by KineTAC disrupts the LRP8–SEPP1 axis, reducing selenoprotein translation, elevating lipid peroxidation, and sensitizing tumor cells to ferroptosis. Schematic was generated using Biorender. All statistics were determined by one-way ANOVA. *P < 0.05. **P < 0.01. ****P < 0.0001. ns, not significant.

To determine whether disrupting the SEPP1–LRP8 axis enhances ferroptotic vulnerability, we treated HCC1143 cells with ferroptosis inducers in the presence of KineTACs. The KineTAC variants markedly sensitized cells to the covalent GPX4 inhibitor RSL3 (13), and this effect was fully rescued by the radical-trapping antioxidant ferrostatin-1 (Fer-1) (49) but not by the pan-caspase inhibitor Z-VAD-FMK (50) (Fig. 5*A*), indicating that KineTAC-mediated cell death is driven by ferroptosis rather than apoptosis. KineTAC treatment also strongly potentiated cell death induced by the additional GPX4 inhibitor ML162 (Fig. 5*B*). In addition, KineTACs sensitized HepG2 liver cancer cells, which express high levels of GPX4 (*SI Appendix*, Fig. S8 *A*–*E*) (51, 52).

We further used an Incucyte-based live cell imaging assay to monitor ferroptotic susceptibility. Pretreatment with 50 nM KineTACs for 72 h sensitized Kelly cells to the system Xc^−^ inhibitor imidazole ketone erastin (IKE) (*SI Appendix*, Fig. S8*F*), which indirectly compromises GPX4 function through depletion of cystine and glutathione. KineTAC treatment also enhanced sensitivity to RSL3 (*SI Appendix*, Fig. S8*G*), and all ferroptosis phenotypes were fully rescued by Fer-1 (*SI Appendix*, Fig. S8 *F* and *G*). The differential magnitude of sensitization across ferroptosis inducers may reflect their distinct mechanisms of action: whereas IKE limits cystine uptake and depletes glutathione over time, RSL3 directly inactivates GPX4, leading to more acute but context-dependent lipid peroxidation responses. Differences in compound permeability, redox buffering capacity, and cell type–specific antioxidant programs could also contribute to the observed variability.

To evaluate lipid peroxidation, HCC1143 cells were treated with 50 nM KineTACs for 72 h followed by 5 h RSL3 exposure. KineTAC1 and tKineTAC2 significantly elevated lipid peroxidation (Fig. 5 *C* and *D*). Similar results were observed in Kelly cells, where all KineTAC treatments enhanced RSL3-induced cell death and lipid peroxidation (Fig. 5 *E* and *F* and *SI Appendix*, Fig. S9*A*). Notably, KineTAC treatment alone was sufficient to trigger lipid peroxidation in Kelly cells (*SI Appendix*, Fig. S9 *B* and *C*), highlighting the intrinsic ferroptotic hypersensitivity conferred by LRP8 loss in MYCN-amplified neuroblastoma (23).

Together, these results establish LRP8 degradation as a generalizable strategy to potentiate ferroptosis across tumor cell types. Mechanistically, targeted degradation of LRP8 disrupts the SEPP1–LRP8 axis, restricting selenium availability for selenoprotein biosynthesis, thereby perturbing lipid homeostasis and promoting lipid peroxidation (Fig. 5*G*).

## Discussion

Ferroptosis sensitivity in cancer is tightly constrained by access to key metabolic inputs, including selenium required for GPX4 synthesis. This dependence creates an opportunity to modulate ferroptosis indirectly by targeting extracellular pathways that control nutrient uptake rather than intracellular enzymes themselves. eTPD is emerging as a powerful modality for antibody-based therapeutics. In this study, we engineered antibodies recognizing two distinct epitopes on LRP8 and assembled them into bispecific LRP8-targeting KineTACs capable of robustly degrading the membrane bound LRP8. By selectively removing this receptor, we demonstrated that extracellular degradation can indirectly downregulate intracellular pathways that depend on selenium availability, providing direct evidence for the functional coupling of the LRP8–GPX4 axis. Whole cell proteomics revealed the high selectivity of KineTAC-mediated degradation. Selenium supplementation restored GPX4 levels, further validating the selenium-dependent nature of this regulatory network. We also found that increasing molecular avidity and valency improves degradation potency, consistent with prior work on other degrading receptors such as folate receptors (44) and transmembrane E3 ligases (53).

Importantly, we show that targeting LRP8 sensitizes tumor cells to ferroptosis, highlighting LRP8 as a promising therapeutic entry point for regulating lipid peroxidation. In contrast, although GPX4 is a central suppressor of ferroptosis, it is also indispensable for normal tissue homeostasis. Systemic inhibition of GPX4 can therefore induce ferroptosis in healthy cells, including those in the kidney (14, 54), potentially narrowing the therapeutic window (55). Moreover, many early GPX4 inhibitors (e.g., RSL3, ML162, and ML210) are covalent compounds that perform well in cultured cells but display poor pharmacokinetics and limited target engagement in vivo (56). High chemical reactivity can promote promiscuous binding to proteins containing reactive cysteine or selenocysteine residues, leading to off-target cytotoxicity that is not directly attributable to ferroptosis.

Our findings suggest that extracellular targeting of LRP8 may offer a safer and more selective strategy to modulate ferroptosis, circumventing the liabilities associated with direct GPX4 inhibition. Consistent with this concept, our LRP8-directed KineTACs effectively sensitize diverse tumor models, including TNBC, liver cancer, and neuroblastoma, to ferroptotic stress. Certain cancers, particularly those with low system Xc^−^ activity, that contributes to the transport of specific selenium species, appear especially dependent on LRP8 and therefore more vulnerable than normal tissues. A prominent example is for MYCN-amplified neuroblastoma (23), in which tumor cells have limited alternative pathways for selenium uptake. Notably, our LRP8-directed KineTACs function both as receptor degraders and as functional SEPP1 blockers, thereby simultaneously reducing selenium uptake and compromising GPX4 expression/activity through a dual mechanism.

As an endocytic member in the LDLR family (57), LRP8 serves as a viral entry receptor (58–60) and potentially undergoes fast internalization, opening opportunities beyond protein degradation. The LRP8 antibodies we developed could be adapted into antibody–drug conjugates carrying GPX4 inhibitors (2, 61), enabling the selective delivery of ferroptosis-inducing agents into LRP8-expressing tumor cells. Together, these findings position LRP8 as a versatile therapeutic node and highlight KineTAC-based extracellular degradation as a compelling approach for sensitizing tumors to ferroptosis in a targeted and potentially safer manner.

## Materials and Methods

### Plasmid Construction.

All the IgGs were constructed in a pcDNA3.4 vector that expresses the light chain and heavy chain, respectively for mammalian expression. For generating bispecific antibodies, the heavy chain variable regions and CXCL12 were cloned into zymework-A mutant Fc (T350V/L351Y/F405A/Y407V) and zymework-B mutant Fc (T350V/T366L/K392L/T394W) sequences respectively (62). Both Fc chains contained additional L234A/L235A/P329G mutations (39) to eliminate Fc effector function for macrophage and NK cell recruitment. For generating the tetravalent KineTAC2, CXCL12 was cloned into the N terminus of 203F4 light chain with (G_4_S)_3_ linkers whereas 203F4 heavy chain was cloned in unmutated Fc. LRP8β antigen was cloned into pFuse vector with IL-2 signal peptide followed by TEV protease, Fc, and Avitag sequence in the C-terminus. The 10 selenocysteines from SEPP1 (Uniprot P49908) were replaced with cysteines, and then cloned into the pcDNA3.4 vector with a C-terminal His10 tag. All the Fabs were constructed in a dual-expression pBL347 vector that expresses the light chain and the heavy chain with the pelB and the stII signal peptides, respectively, for the periplasm expression.

### Cell Lines.

MDA-MB-231 and HepG2 cells were cultured in DMEM (ThermoFisher Scientific) with 10% fetal bovine serum (FBS) and 1% penicillin–streptomycin. HCC1143, Kelly, SK-N-DZ and HT-1080 cells were cultured in RPMI-1640 (ThermoFisher Scientific) with 10% FBS and 1% penicillin–streptomycin. HCC1143 LRP8 vehicle (Cas9) cells and KO3 (24) cells were supplemented with 100 μg/mL hygromycin (Sigma-Aldrich). Expi293 cells were cultured in Expi293 expression medium (ThermoFisher Scientific).

### Phage Display.

Phage selection was done as described previously (32). In brief, library E from University of Chicago and UCSF library were incubated with streptavidin-coated magnetic beads preconjugated with biotinylated Fc protein to remove nonspecific binders. Unbounded phage were then incubated with streptavidin-coated magnetic beads preconjugated with biotinylated LRP8β-TEV-Fc antigens. After 4 washes, antigen-bound phage were eluted from beads by incubating with 1 μM TEV protease for 20 min. In total, four rounds of selections were performed with a decreasing concentration of LRP8β-TEV-Fc-antigen (1000, 50, 20, 10 nM). From round 3, the phage library was first enriched by protein A magnetic beads to deplete nondisplayed or truncated Fab phage before each round of the selection.

### Phage ELISA.

384-well Maxisorp plates were coated with Neutravidin (10 μg/mL) overnight at 4 °C and subsequently blocked with BSA (2% w/v) for 1 h at RT. 20 nM biotinylated LRP8β-TEV-Fc, BSA, or empty Fc antigens were captured on the NeutrAvidin-coated wells for 30 min followed by the addition of 1:5 diluted single-colony phage for 1 h. For competition, 20 nM of soluble LRP8β-TEV-Fc antigen was added to the diluted phage and incubated for 1 h. The bound phage was then detected by a horseradish peroxidase (HRP)-conjugated anti-M13 phage antibody (Sino Biological). The ELISA plates were washed three times after each incubation, and antibody binding was detected by TMB substrate (VWR) and read at 450 nm.

### Protein Expression.

IgGs and antigen were expressed in Expi293 cells. For 30 mL transfection, 24 μg of DNA was added to 3 mL of OptiMEM, followed by 24 μL of FectoPro transfection reagents. After 10 min of incubation, 27 mL of Expi293F cells at 3 millions/mL were added and shake at 37 °C. Biotinylated antigens were expressed in Expi293 cells stably expressing BirA, and 3 mL of 4 mM biotin was added at transfection. On the second day, 300 μL of 300 mM valporic acid and 270 μL of 45% glucose were added to the cells. After 5 d, cultures were harvested, centrifuged at 4,000 g for 20 min, and clarified through a 0.45-μm Steriflip filter. Supernatants were then incubated with Sepharose A resin (Cytiva) for 2 h, proteins were then eluted by 0.1 M acetic acid and neutralized by Tris pH 11. Proteins were buffer-exchanged at least twice into PBS using Amicon concentrators (Millipore Sigma). For His-tagged proteins, culture supernatants were incubated with HisPur™ Ni-NTA resin (Thermo Fisher Scientific) for 2 h at 4 °C and eluted with 500 mM imidazole, followed by PBS buffer exchange using Amicon concentrators. Fabs were expressed in *Escherichia coli* C43 (DE3) Pro+ grown in an optimized TB autoinduction medium at 37 °C for 6 h, cooled to 30 °C for 18 h. Cells were harvested by centrifugation and lysed using B-PER lysis buffer (ThermoFisher Scientific). The lysate was incubated at 60 °C for 20 min and centrifuged to remove the inclusion body. The Fabs were purified by Sepharose A resin via affinity chromatography and buffer exchanged in PBS for further characterization. Purity and integrity of all proteins were assessed by SDS–PAGE (Thermo Fisher Scientific).

### Recombinant Protein ELISA.

384-well Maxisorp plates were coated with neutravidin (10 μg/mL) or 6× Histag antibody (Invitrogen, Cat# MA1-21315, 2 μg/mL) overnight at 4 °C and subsequently blocked with BSA (2% w/v) for 1 h at RT. 20 nM of antigens were captured onto precoated wells for 1 h. Recombinant LDLR, LRP2, and LRP8 proteins were purchased from ACROBiosystems. For polyspecificity ELISAs, the autoantigens cardiolipin (Sigma, 50 μg/mL), insulin (Sigma, 1 μg/mL), lipopolysaccharide (LPS, InvivoGen, 10 μg/mL), and single-stranded DNA (ssDNA, Sigma, 1 μg/mL), were directly coated onto plates overnight 4 °C. After three washes, serially diluted Fabs were added to the plates and incubated for 1 h at RT. After three washes, 1:5,000 diluted peroxidase-anti-human IgG (H+L) (Jackson ImmunoResearch) were added to the plates and incubated for 30 min. After three times of washing, antibody binding was detected by TMB substrate (VWR), quenched by 1 M phosphoric acid, and read at 450 nm.

### Biolayer Interferometry.

BLI experiments were performed at room temperature using an Octet RED384 instrument (ForteBio). 20 nM biotinylated antigen-Fc was immobilized to an optically transparent SA biosensor (ForteBio). Different concentrations of monomeric Fab antibodies or SEPP1-Cys in kinetics buffer (PBS, 0.05% Tween-20, 0.2% BSA) were used as the analyte in a 384-well microplate (Greiner Bio-One). Because the Fc format presents antigens bivalently on the sensor surface, affinities (K_D_s) were best fit with a 1:1 binding model and calculated by a global fit analysis using the Octet RED384 Data Analysis HT software.

### Epitope Binning by BLI.

Anti-LRP8 antibodies were binned into epitope specificities using an Octet RED384 system. 20 nM of biotinylated LRP8β-Fc antigens were captured using streptavidin biosensors (Fortebio). After antigen loading, a saturating concentration of antibodies or SEPP1 (200 nM) was added for 10 min. Competing concentrations of antibodies or SEPP1 (50 nM) were then added for 5 min to measure binding in the presence of saturating antibodies. All incubation steps were performed in PBS/0.05% Tween-20/0.2% BSA.

### SEPP1 Uptake Assay.

SEPP1-Cys was labeled using the LysoLight Antibody Labeling Kits (Invitrogen, Cat# L36003) following the manufacturer’s instructions. Briefly, SEPP1 were labeled with LLDR with a molar ratio of 1:6 in the presence of 100 mM sodium bicarbonate (pH 8.4) for 2 h at RT. Labeled SEPP1 was then purified with 7k Zeba dye and biotin removal columns (Thermo Scientific, Cat#A44297). HCC1143 cells were seeded at 5,000 cells/well on a 96-well polystyrene tissue culture treated plate (Corning, Cat#3596). The next day, the media was removed and cells were serum starved for 12 h. Next, cells were pretreated with 200 nM anti-LRP8 Fabs or PBS for 30 min at 37 °C respectively, followed by 50 nM of SEPP1-LLDR treatment with or without 0.5 μM cytochalasin D. Cells were then imaged by Incucyte (Sartorius) every 2 h. Internalization was calculated by total integrated intensity (NIRCU × μm^2^/image) on the Incucyte software.

Additional materials and methods in this study are detailed in *SI Appendix*, which also describes the experimental procedures for flow cytometry, western blot, proteomics, and ferroptosis assays.

## Supplementary Material

Appendix 01 (PDF)

## Data Availability

All raw and searched mass spectrometry data have been deposited in ProteomeXchange Consortium via the MassIVE partner repository with the dataset identifier PXD078567 (63). All other data are included in the manuscript and/or *SI Appendix*.
