# Supplementary material for: Targeted extracellular degradation of LRP8 promotes ferroptosis in cancer cells
Source: Proc Natl Acad Sci U S A. 2026 Sep 30;123(40):e2616933123. doi: 10.1073/pnas.2616933123 (PMC13643147; doi:10.1073/pnas.2616933123)
Supplement: Supplementary file 1 [file pnas.2616933123.sapp.pdf]

PNAS

Supporting Information for

Targeted extracellular degradation of LRP8 promotes ferroptosis in cancer cells

Fangzhu Zhao[1], Alex Inague[2,3], Trenton M. Peters-Clarke[1], Yifei Chen[1], Snehal D. Ganjave[1], Yun Zhang[1], Kun Miao[1], Zi Yao[1], Yan Wu[1], Madison K.C. Seto[1], Kevin K. Leung[1], James A. Olzmann[2,3], James A. Wells[1,4*]

[1]Department of Pharmaceutical Chemistry, University of California San Francisco, San Francisco, CA, USA
[2]Department of Molecular and Cell Biology, University of California Berkeley, Berkeley, CA, USA.
[3]Department of Metabolic Biology and Nutrition, University of California Berkeley, Berkeley, CA, USA
[4]Department of Cellular & Molecular Pharmacology, University of California San Francisco, San Francisco, CA, USA

*Correspondence to: James A. Wells
Email: jim.wells@ucsf.edu

This PDF file includes:

Materials and Methods
Figures S1 to S9
SI References

Materials and Methods

Structural modeling and energy analysis

Structural models of the SEPP1–LRP8 complex were generated using AlphaFold3 (https://alphafoldserver.com/). The amino acid sequences of human LRP8β and SEPP1 were used as input, with selenocysteine residues in SEPP1 substituted with cysteine to enable modeling. Default multimer settings were applied, and top-ranked models were selected based on predicted alignment error (PAE) and confidence scores. Predicted complexes were visualized and analyzed using ChimeraX. To evaluate binding energetics, AlphaFold-derived models were subjected to Rosetta-based scoring (Rosetta 3.71). Structures were relaxed using the FastRelax protocol, and interface energies (ΔG) were calculated using the InterfaceAnalyzer module with default parameters. Residue-level contributions and interface metrics were extracted to assess key determinants of SEPP1–LRP8 binding.

Flow cytometry

Cells were harvested by 0.05% Trypsin-EDTA and centrifuged at 400 g for 5 min. Pellets were washed once with PBS + 1% BSA. Cells were incubated with Fab or IgG antibodies in PBS + 1% BSA for 30 min at 4 °C. After two washes, cells were then stained with Alexa fluor 647 goat anti-human IgG (H+L) (Invitrogen, Cat#A-21445, 1:1000) for 30 min at 4 °C. After two washes, cells were resuspended in PBS + 1% BSA. Flow cytometry was performed using a CytoFLEX cytometer (Beckman Coulter, v.2.3.1.22) and CytoExpert software (v.2.3.1.22). Data were analyzed with FlowJo (v.10.8.0).

Chemicals and reagents

1S,3R-RSL3 (referred to as RSL3) (Cat#HY-100218A), MG132 (Cat#HY-13259), and Z-VAD-FMK (Cat#HY-16658B) were purchased from MedChemExpress. Cytochalasin D (Cat#11330), ML162 (Cat#20455), IKE (Cat#27088), and Ferrostatin-1 (Cat#17729), were all purchased from Cayman Chemical Company. Bafilomycin A1 (Cat#sc-201550A) was purchased from Santa Cruz Biotechnology. L-Selenocystine (Cat#545996), sodium selenite (Cat#S5261), acetonitrile (HPLC grade), H_2O (HPLC grade), and formic acid (LC-MS grade) were obtained from Sigma-Aldrich.

Degradation experiments

Cells were plated onto 6- or 12-well plates (Corning) and grown to ~70% confluency before treatment. On the next day, cell culture medium was aspirated, various concentrations of antibodies in 1-2 mL of culture medium were then added to each well. Cells were incubated for 24 h or 48 h or 72 h at 37 °C prior to western blotting experiments. For inhibitor assays, cells were pretreated with 0.5 μM Bafilomycin A, 0.5 μM MG132, or 0.5 μM Cytochalasin D for 1 h, respectively prior to antibody treatment.

Western blotting

Cells were lifted with versene (ThermoFisher Scientific), washed twice with PBS, and then lysed in RIPA buffer (EMD Millipore) with cOmplete mini protease inhibitor cocktail (Sigma-Aldrich) for 20 min on ice. The cell lysates were then centrifuged at 20,000 g for 10 min at 4 °C to remove any cell debris. Soluble protein concentrations were quantified by Rapid Gold BCA Protein Assay Kit (Pierce). Lysates were mixed with 4× Nupage LDS Sample Buffer (Invitrogen) and 2-mercaptoethanol, and then run on NuPAGE™ 4-12% Bis Tris Protein Gels (ThermoFisher Scientific). Proteins were transferred to polyvinylidene difluoride membranes using the iBlot2 Western Blotting Transfer System (Thermo

Scientific). Membranes were blocked with TBS + 5% BSA + 0.5% Tween (PBST) for 30 min, and stained with primary antibodies overnight. After three washes, membranes were stained with secondary antibodies for 1 h at RT. After three washes, membranes were imaged with a LICOR imager (LI-COR Biosciences) or the ChemiDoc MP imaging system (BioRad). For HRP detection, Supersignal West Pico PLUS Chemiluminescent Substrate (Thermo Scientific, Cat# 34577) was added to the membrane. Antibodies used included rabbit anti-human ApoER2 (Abcam, Cat#ab108208, 1:1,000), rabbit anti-human GPX4 (Abcam, Cat#ab125066, 1:2,000), rabbit anti-human GPX1 (Abcam, Cat#ab22604, 1:1000), mouse anti-human SELENOM (Santa Cruz, Cat#sc-514952, 1:1000), mouse anti-human TrxR1 (Santa Cruz, Cat#sc-28321, 1:1000), mouse anti-human α-tubulin (Cell Signaling Technology, 3873S, 1:3,000), mouse anti-human β-actin (Cell Signaling Technology, 3700S, 1:3,000), IRDye 800CW goat anti-rabbit IgG (LI-COR Biosciences, Cat#926-32211), IRDye 680RD goat anti-mouse IgG (LI-COR Biosciences, Cat#926-68070, 1:5000), peroxidase goat anti-rabbit IgG (H+L) (Jackson ImmunoResearch, Cat# 111-035-144, 1:5000).

Proteomics sample preparation

0.5 M Kelly cells were seeded onto 6-well plates in RPMI1640 medium supplemented with 10% dialyzed FBS and 1% penicillin–streptomycin overnight at 37 °C. The next day, media were removed, and 50 nM of antibodies were added to cells with or without 50 nM of L-selenocystine. After 48 h of treatment, cells were lifted with versene, and transferred to protein LoBind Eppendorf tubes. Cell pellets were washed three times with cold PBS, and flashed frozen until proteomic sample preparation.

Proteins were digested and desalted using the Preomics iST kit according to the manufacturer's specifications (PreOmics, Cat#P.O.00027). Cell pellets were resuspended in the LYSE solution and sonicated with probe at 20% amplitude for 1 min (2 s on/4 s off). Cell lysates were clarified at 16,000 x g for 10 min and supernatant was incubated at 95 °C for 10 min for cysteine reduction and alkylation. DIGEST enzyme pellets were reconstituted in 210 µL RESUSPEND solution. Samples were cooled to room temperature and combined with 50 µL DIGEST solution. Incubation was performed at 37 °C for 4 hr while shaking. After the digestion, quenching of enzymatic activity was performed by adding 100 µL STOP solution to digested samples to a final pH below 2.0. Samples were spun through a PreOmics desalting column at 3,800 x g for 2 min. Samples were then washed with 200 µL WASH 1 and 200 µL WASH 2 each at 3,800 x g for 1 min. Peptides were eluted using 100 µL ELUTE solution in a new low-bind tube at 3,800 x g for 2 min. Desalted samples were dried down via SpeedVac and resuspended in 0.2% formic acid (FA) in water. Peptide concentration was determined using a Pierce Quantitative Colorimetric Peptide Assay (Thermo Fisher Scientific, Rockford, IL) before liquid chromatography–tandem mass spectrometry analysis.

Liquid chromatography

Liquid chromatography was performed using either a Vanquish Neo UHPLC system (Thermo Fisher Scientific), configured in a direct injection format, or using a nanoElute 2 UHPLC (Bruker), configured in a trap-and-elute format. Peptides were separated on the Vanquish Neo with an Aurora Ultimate C18 120 Å, 1.7 µm, 75 µm x 25 cm UHPLC column (Ion Opticks). During LC separations, mobile phase A (MPA) was 0.1% FA in water and MPB was 80% ACN in water with 0.1% FA. For profiling of the proteome, a 90-min gradient ramped, at a flow rate of 250 nL/min, from 0-1% MPB from 0 – 1 min, 1-28% MPB from 1 – 80 min, 28 – 58% MPB from 80 – 89 min, 58 – 99% MPB from 89 – 90 min, and held at 99% MPB to 100 min before the column was washed and re-equilibrated at 0% MPB for

three column volumes. During peptides separations, the LC column was held at 50 °C. On the timsTOF platform, peptides were separated using a nanoElute 2 with a 100 Å, 5 µM, 5 mm x 0.3 mm trap cartridge (Thermo Fisher) in line with a PepSep C18 100 Å, 1.5 µM, 25cm x 150 µM column on a timsTOF Pro 2 with a Captive Spray source and a nanoElute line (Bruker, Hamburg, Germany). During LC separations, MPA was 0.1% FA in water and MPB was ACN with 0.1% FA. A 62-min gradient ramped, at a flow rate of 500 nL/min, from 2-28% MPB from 0 – 60 min, 28-32% MPB from 60 – 62 min, 32 – 95% MPB from 62 – 62.5 min, and held at 95% MPB to 70 min before the column was washed and re-equilibrated at 0% MPB for 8 min. During peptide separations, the LC column was held at 50 °C.

Mass spectrometry
Data independent acquisition (DIA) was performed either on a Thermo Scientific™ Orbitrap Eclipse™ Tribrid™ hybrid mass spectrometer system (Thermo Fisher Scientific, San Jose, USA) or on a timsTOF Pro 2 (Bruker, Hamburg, Germany). On the Orbitrap Eclipse, precursors were ionized using electrospray ionization at 2 kV with respect to ground. The inlet capillary was held at 275 °C, and the ion funnel RF was held at 60%. During DIA experiments, all MS1 survey scans were acquired at a resolving power of 60,000 in the Orbitrap analyzer with a scan range of m/z 380 – 980, maximum injection time of 50 ms, and AGC target of 1,000,000 charges. DIA scans were acquired in the Orbitrap analyzer at resolution 15,000 with precursor mass range set to m/z 380-980. An isolation width of 14 Th with 1 Th window overlap created 42 bins across the m/z range. HCD normalized collision energy (NCE) was set to 30%. MS/MS scans required an injection time of 22 ms or 100,000 charges. For data acquisition on the timsTOF platform, ions were generated in positive mode with the capillary set to 4500 V. diaPASEF windows were generated between 0.69 V•s/cm2 to 1.29 V•s/cm2 in the 1/K0 dimension and between m/z 273 to 1173 in the mass dimension. A mass width of 25 m/z was used, yielding 36 steps per cycle. Ramp time was set to 120 ms, ramp rate was 7.93 Hz, and cycle time estimate was 1.26 s. A scan range of m/z 100 to 1700 was used.

Data analysis
DIA .raw and .d files were processed using Spectronaut (20.2.250922.92449). Settings denote proteolytic cleavage C-terminal to lysine and arginine not before proline with up to 2 missed cleavages. A FASTA file containing the reviewed human proteome and isoforms was downloaded from UniProt on April 9, 2024 and imported into Spectronaut, where mutation-based decoy sequences were generated for false discovery rate estimation. Peptide length of 7 to 52 amino acids was specified. Alkylation of cysteines was fixed while oxidation of methionine and protein N-terminal acetylation were variable modifications. Data was processed in library-free directDIA mode. Precursors, peptide identifications, and proteins were filtered to maintain 1% FDR. Proteins identified with only one peptide were filtered out of the final dataset. Data were further analyzed in RStudio (2025.09.2) using Limma differential analysis.

BODIPY 581/591 C11 assay
Cells seeded in a 12-well plate were treated with KineTACs or controls for 24 h 37°C, followed by 200 nM RSL3 treatment for 5 h and washed once with DPBS containing calcium and magnesium. Cells were detached from the plate with versene and incubated with 5 µM BODIPY 581/591 C11 (Invitrogen, Cat#D3861) at 37°C for 10 min. After two washes, cells were then stained with LIVE/DEAD™ fixable NIR-IR dye (Invitrogen, Cat# L10119). After two washes, cells were resuspended in PBS + 1% BSA. Flow cytometry

was performed using a CytoFLEX cytometer (Beckman Coulter, v.2.3.1.22) and CytoExpert software (v.2.3.1.22). Data were analyzed with FlowJo (v.10.8.0).

Cell viability and death analysis

For cell viability analysis, the CellTiter-Glo assay (Promega) was performed according to the manufacturer's instructions. Briefly, 5000 cells/well were seeded on a 96-well polylysine-coated white plate (Corning, Cat#3917). The next day, media was aspirated, 100 µL fresh medium containing 100 nM of KineTACs or control antibodies were added to the cells for 24 h at 37 °C. Then 100 µL of ferroptosis compounds at various concentrations were added to the cells, with or without 1 µM Fer-1 or 5 µM Z-VAD-FMK. After 24 h of compound treatment, cell culture media was removed and viability was measured using CellTiter-Glo Reagent (Promega). The plates were incubated at room temperature for 10 min, and the luminescent signal was measured on the Spark microplate reader (Tecan).

Cell death was also analyzed using the IncuCyte S3 live-cell imaging system (Essen Bioscience) following a modified protocol adapted from Zhang *et a*l(5). Prior to treatment with ferroptosis-inducing agents (RSL3 or ML-210), Kelly cells were seeded at 1,500 cells per well in triplicate in black 384-well plates (Greiner, Cat. #781091) and pretreated with 50 nM KineTAC1, 50 nM tKineTAC2, or PBS for 72 h in an incubator set to 37 °C and 5% CO_2.

For Kelly cells, a separate drug plate was prepared by titrating different concentrations of RSL3 or ML-210 in a low-adhesion 384-well plate (PlateOne, Cat. #1884-2410). Medium containing 60 nM SYTOX Green Dead Cell Stain, with or without 2 µM Fer-1, was added to the corresponding wells. Plates were homogenized using a Bioshake 3000 ELM orbital shaker at 2,400 rpm for 45 s. The drug-containing medium was then carefully transferred onto the pre-seeded cells (black 384-well plates), resulting in final concentrations of 30 nM SYTOX Green and 1 µM Fer-1. Plates were immediately placed in the IncuCyte S3 imaging system within an incubator maintained at 37 °C and 5% CO_2. Phase-contrast and green fluorescence images were acquired every 2 h over 24- or 72-h periods. Cell death was quantified as the ratio of SYTOX Green–positive objects per image to total cell area (confluence %) using IncuCyte S3 image analysis software (Essen Bioscience). For each treatment condition, these ratios were plotted over time, the area under the curve (AUC) was calculated, and mean AUC values were plotted as a function of drug concentration using Prism (GraphPad).

Data availability

All raw and searched mass spectrometry data have been deposited to the ProteomeXchange Consortium via the MassIVE partner repository with the dataset identifier PXD078567.

Figures S1 to S9

A

Ab ID	LC3	HC1	HC2	HC3
203F1	FGWSYLI	LSYYSI	SIYPYSGYTS	DHRYYDYAYYPGRYWGGF
203F2	GYYLI	LSYSSM	SIYSSYGYTS	GWASWDYYAARYGM
203F3	YWSYAPF	VSYSSI	SIYPYSGSTY	HEWYGSYYYGYYQYGL
203F4	YSSYSHYLI	ISSSYI	SIYSYYGSTY	HGAM

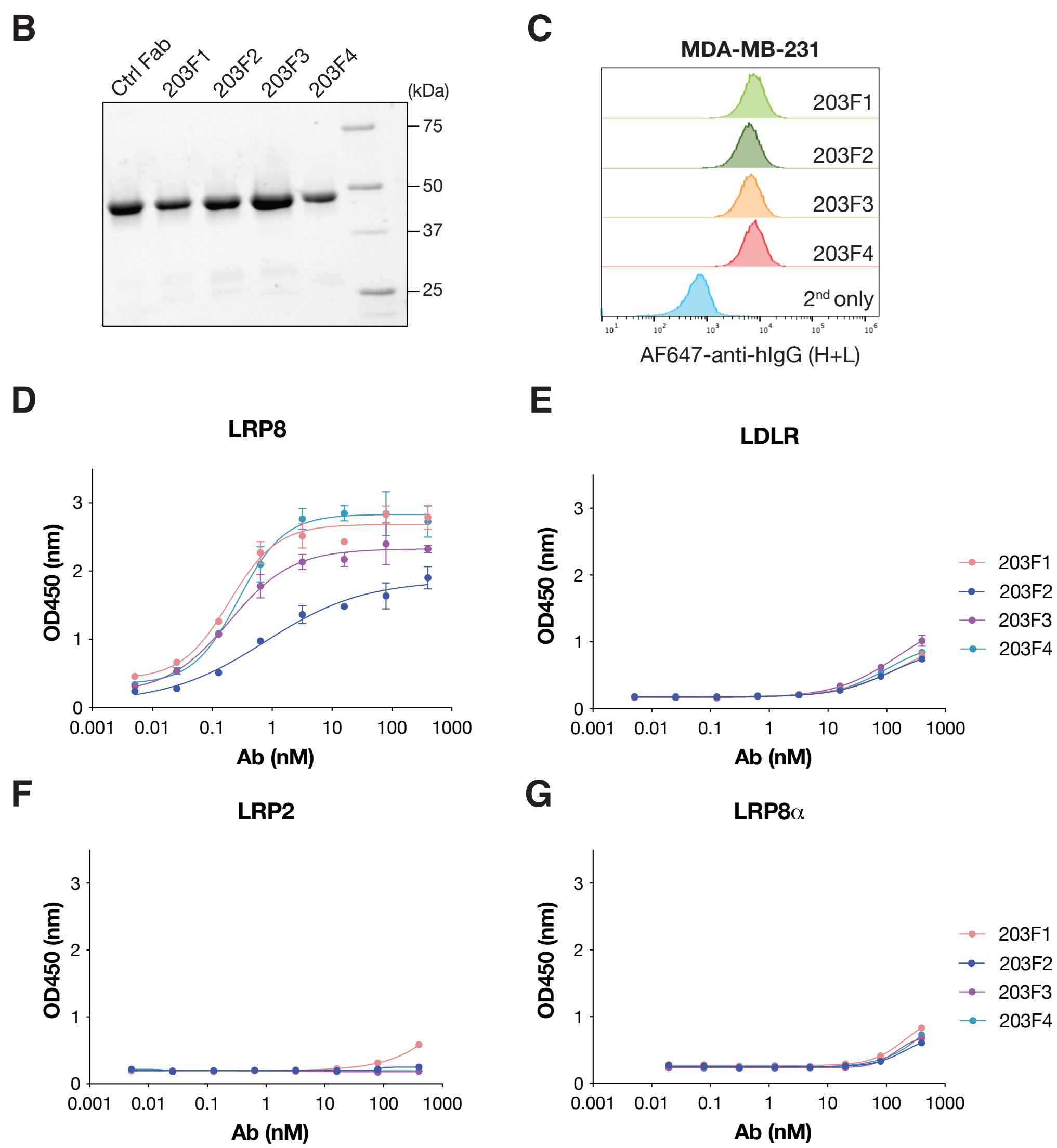

Fig. S1. Characterization of LRP8-specific antibodies. (A) Complementarity-determining region (CDR) sequences of LRP8 antibody heavy chain and light chain. **(B)** SDS-PAGE analysis of recombinant LRP8 Fabs. **(C)** Flow cytometry analysis revealed binding of Fabs against MDA-MB-231 cells with endogenous LRP8 expression. Cells were stained with 20 nM LRP8 Fabs for 30 min, followed by AF647 conjugated anti-human IgG (H+L) antibody staining for 15 min. Data is representative from two independent experiments. **(D-G)** ELISA binding of LPR8 Fabs against recombinant **(D)** LRP8, **(E)** LDLR, **(F)** LRP2, and **(G)** LRP8α. Binding was detected by peroxidase-conjugated goat anti-human IgG (H+L) antibodies. Each sample was tested in duplicate and repeated for at least two independent experiments. Error bars represent standard deviation.

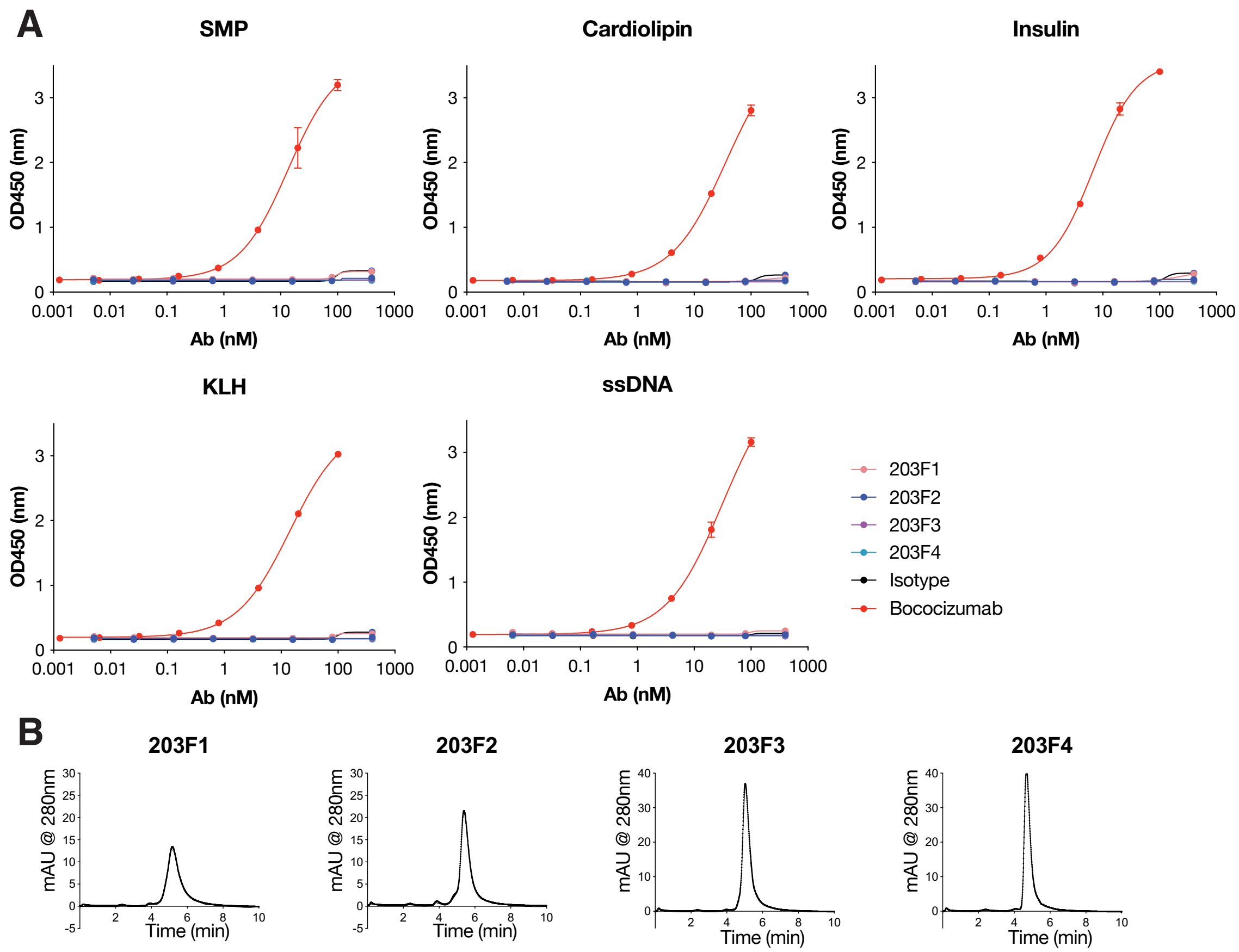

Fig. S2. Polyspecific analysis of LRP8 Fabs. (A) Polyspecific reagent (PSR)(1) ELISA binding of recombinant LPR8 Fabs indicated minimal cross-reactivity to a panel of autoantigens, including solubilized membrane proteins (SMPs), cardiolipin, insulin, keyhole limpet hemocyanin (KLH), and single-stranded DNA (ssDNA). The polyreactive antibody Bococizumab(2) was used as a positive control, while anti-SARS-CoV-2 receptor binding domain (RBD) antibody CC12.1(3) served as a negative control. Binding was detected with peroxidase-conjugated goat anti-human IgG (H+L) antibodies. Data are mean ± SD of two biological experiments. (B) Analytical size exclusion chromatography showing that LRP8 Fabs were monodispersed with regular column retention times.

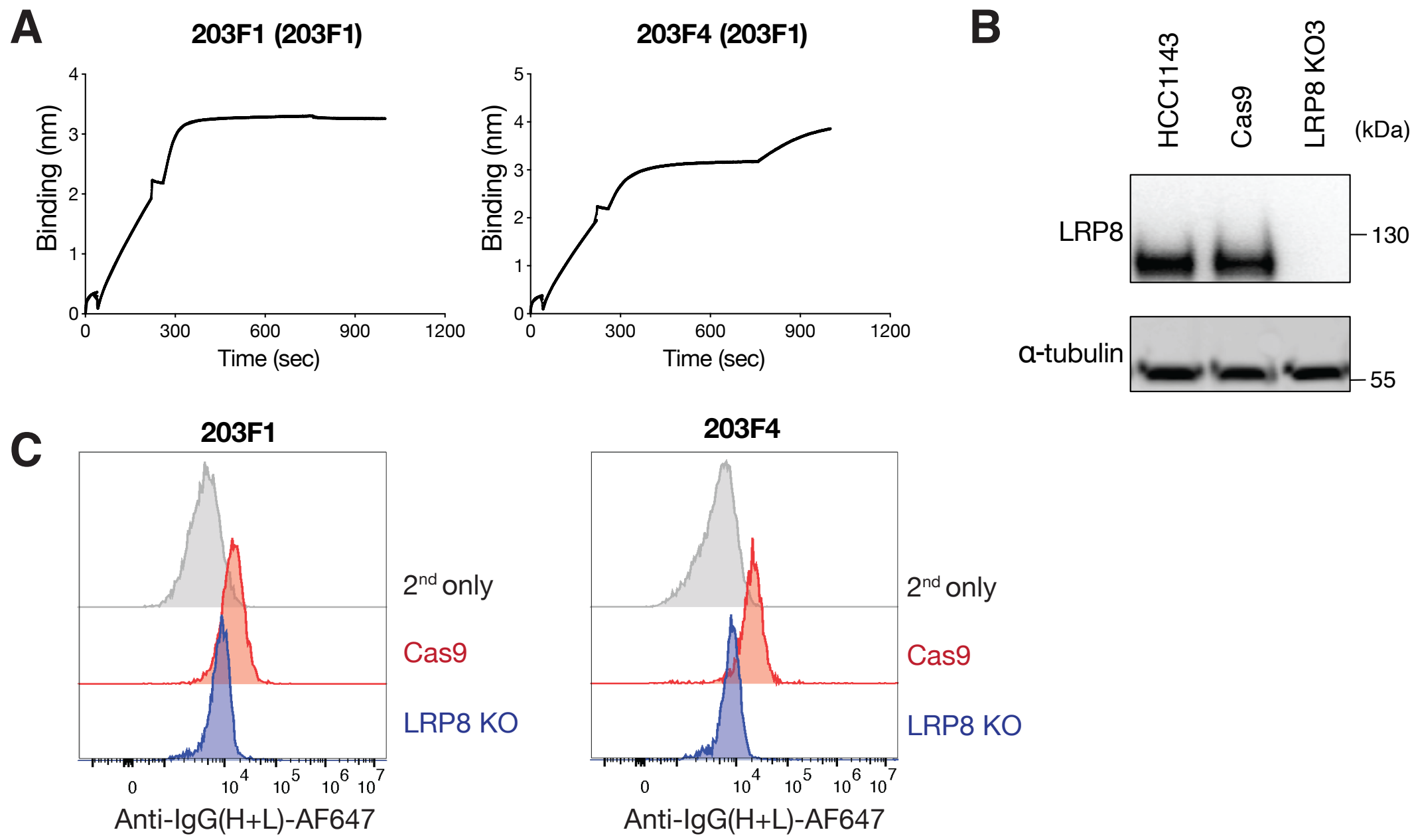

Fig. S3. Epitope specificity and selectivity of LRP8 antibodies. (A) Epitope binning revealed two different Fab binding epitopes on LRP8. Biotinylated LRP8β was captured using a streptavidin biosensor and a saturating concentration (200 nM) of 203F1 or 203F4 Fab were incubated for 10 min followed by incubation with 50 nM of the competing 203F1 Fabs for 5 min. **(B)** Western blot showed LRP8 knockout on HCC1143 cells. **(C)** Flow cytometry analysis revealed Fab binding to LRP8 positive HCC1143 sgRNA control (Cas9) cells but not LRP8 knockout (KO) cells(4). Cells were stained with LRP8 antibodies for 30 min, followed by AF647 conjugated anti-human IgG (H+L) antibody staining for 15 min. Data is representative from two independent experiments.

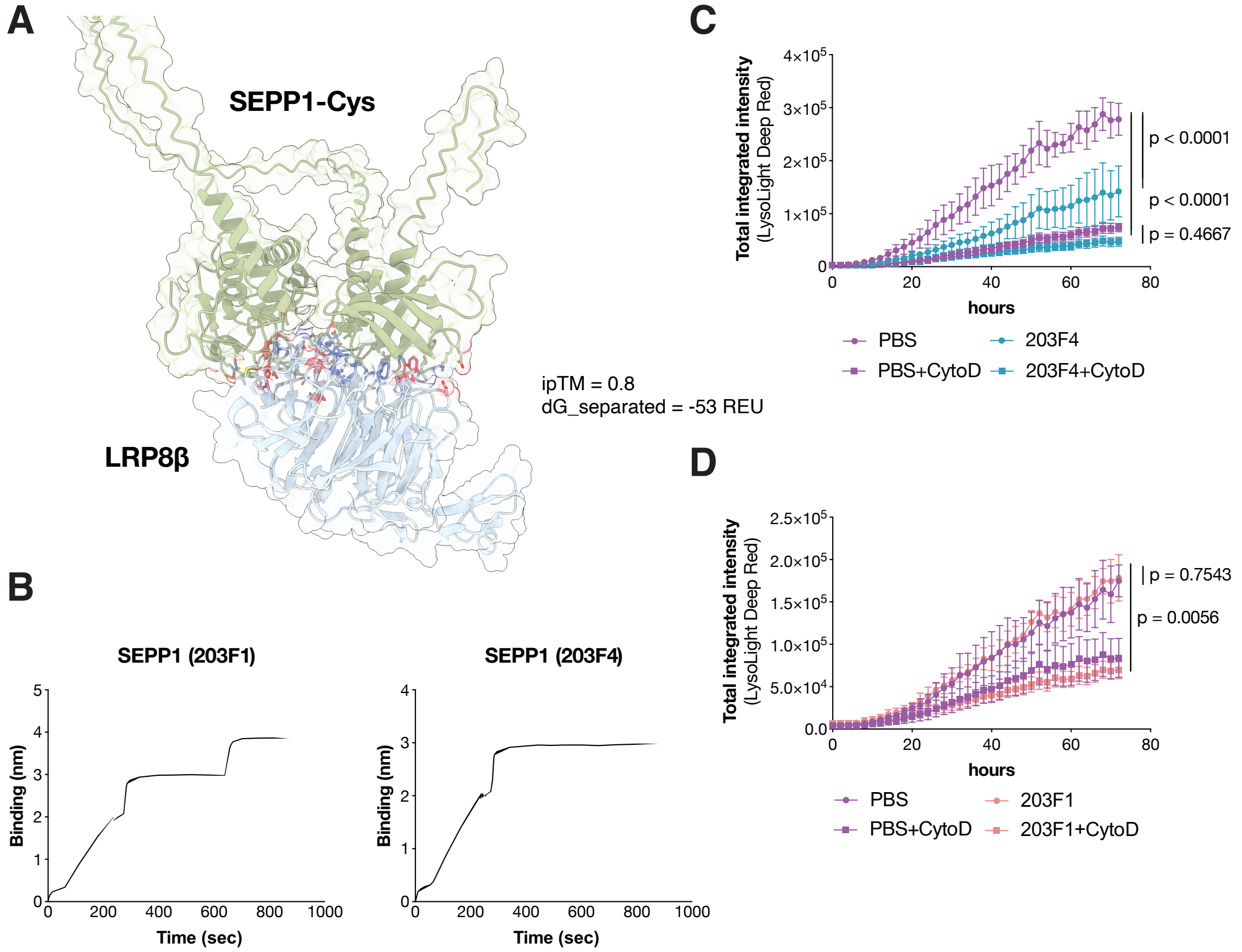

Fig. S4. 203F4 blocks selenoprotein P uptake. (A) AlphaFold3 and Rosetta energy prediction of LRP8β interaction with SEPP1-Cys. Predicted complexes were visualized and analyzed using ChimeraX with a high-confidence interface (ipTM = 0.80). To evaluate binding energetics, AlphaFold-derived models were subjected to Rosetta-based scoring (dG_separated = -53 REU). **(B)** Epitope binning of SEPP1-Cys and LRP8 Fabs revealed two different epitopes on LRP8β. Biotinylated LRP8β was captured using a streptavidin biosensor and SEPP1-Cys at a concentration of 200 nM was incubated for 10 min followed by incubation with 50 nM of the competing LRP8 Fabs for 5 min. All incubation steps were performed in 1x PBS + 0.05% Tween + 0.2% BSA at room temperature. **(C-D)** Internalization of SEPP1-Cys in HCC1143 cells with or without macropinocytosis inhibition. SEPP1-Cys was labeled with the lysolight deep red dye (LLDR) to track lysosomal trafficking. Cells were pre-incubated with 200 nM 203F4 Fab **(C)** or 203F1 Fab **(D)** for 30 min, in the presence or absence of 0.5 μM cytochalasin D (CytoD), followed by treatment with 50 nM SEPP1-Cys-LLDR. Images were captured every 2 h for 72 h on the Incucyte. Total integrated intensity was calculated by NIRCU x μm^2/image. Data represents mean ± SD from three biological replicates. Statistics were calculated by one-way ANOVA and Holm-Sidak multiple comparison tests.

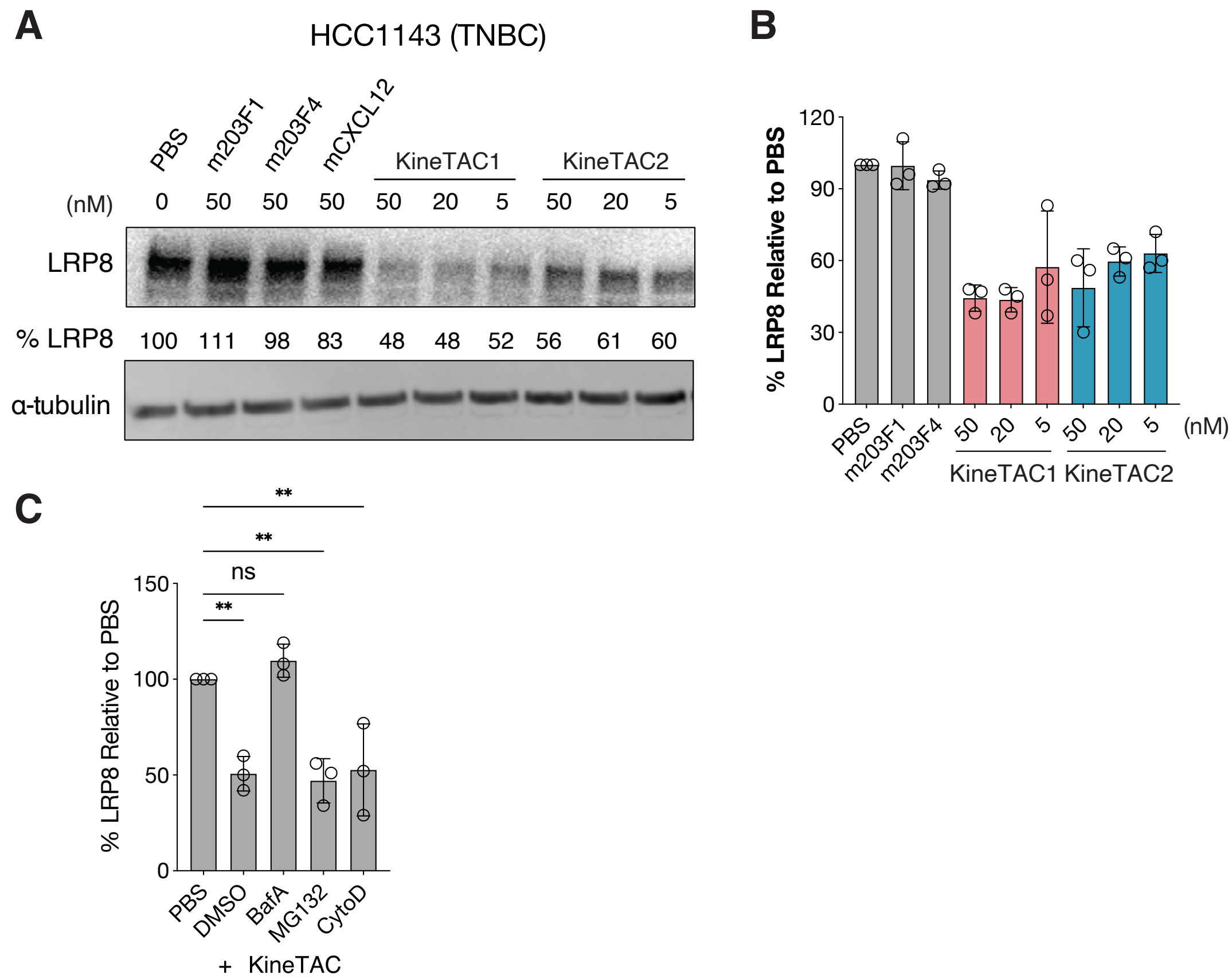

Fig. S5. LRP8 degradation is lysosomal dependent. (A-B) KineTAC-mediated LRP8 degradation in triple-negative breast cancer (TNBC) HCC1143 cells. Cells were treated with KineTAC for monomeric control antibodies (m203F1, m203F4) for 24 h. **(A)** Western blotting showed KineTAC-mediated degradation of LRP8 whereas monomeric antibody did not. **(B)** Percent LRP8 levels were quantified by ImageJ relative to the PBS control. Data represents mean ± SD from three biological replicates. **(C)** Cells were pretreated with either 500 nM Bafilomycin A (BafA), 500 nM MG132, or 500 nM Cytochalasin D (CytoD) for 1 h followed by 24 h treatment with 50 nM KineTAC. Percent LRP8 levels were quantified by ImageJ relative to the PBS control. Error bars represented standard deviations for three biological replicates. Statistics were calculated by one-way ANOVA. $^{**}P < 0.01$. ns, not significant.

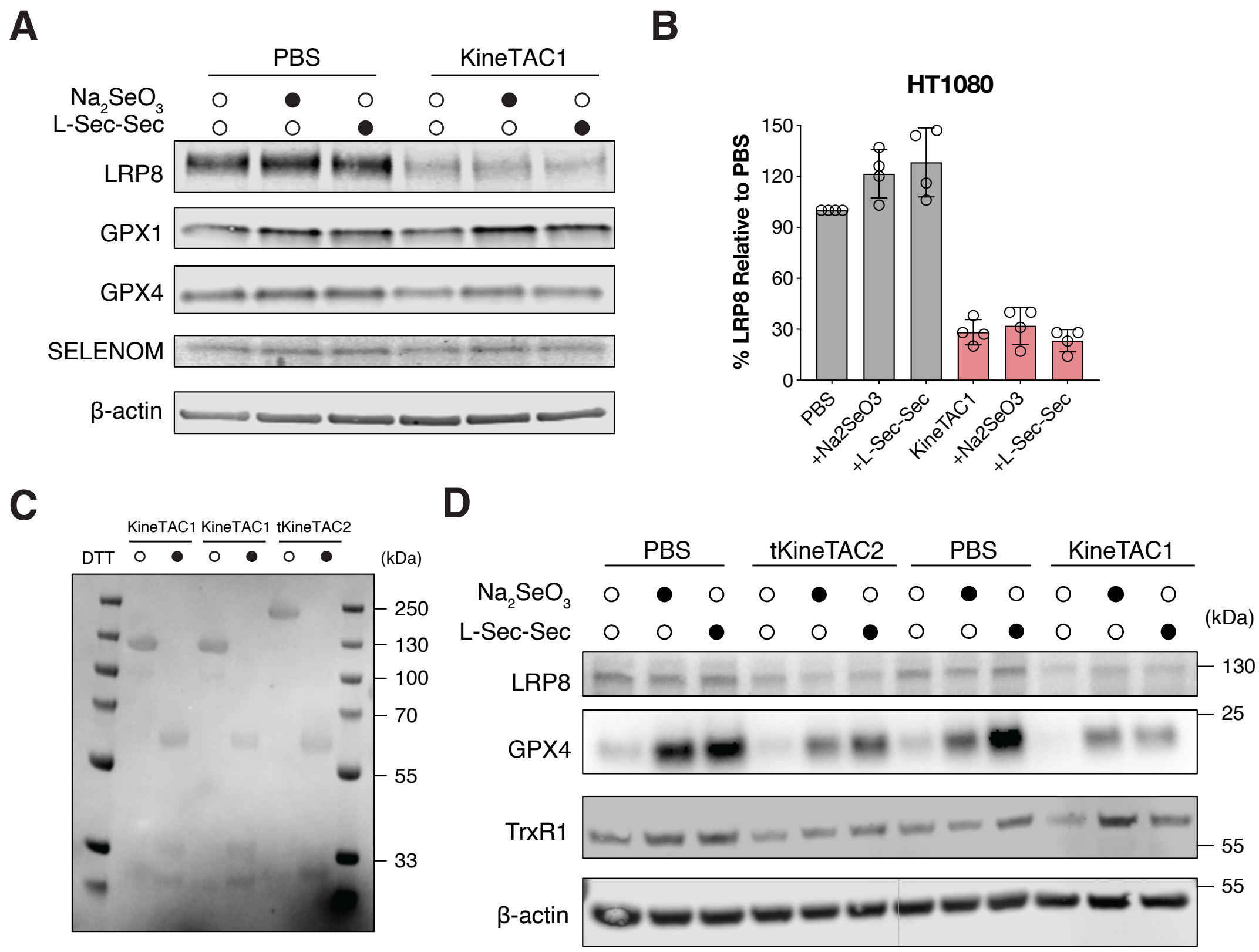

Fig. S6. LRP8 degradation reduces selenoprotein levels. (A-B) Western blotting analysis of HT-1080 cells treated with KineTAC1 in the presence or absence of selenium supplementation. 50 nM KineTAC1 treatment for 72 h efficiently degraded LRP8. Sodium selenite or L-selenocystine treatment didn't affect LRP8 degradation but rescued selenoprotein abundance. Percent LRP8 levels were quantified by ImageJ relative to the PBS control. Data represents mean ± SD from four biological replicates. **(C)** SDS-PAGE analysis of recombinant KineTAC1, KineTAC2, and tKineTAC2. Antibody samples were reduced with dithiothreitol (DTT) and denatured by boiling at 95 °C for 5 min. **(D)** Western blotting analysis of Kelly cells treated with KineTAC1 or tKineTAC2 for 72 h in the presence or absence of selenium supplementation. Whole cell lysates were blotted with LRP8, GPX4, TrxR1 and β-actin. Data are representative from at least three independent experiments.

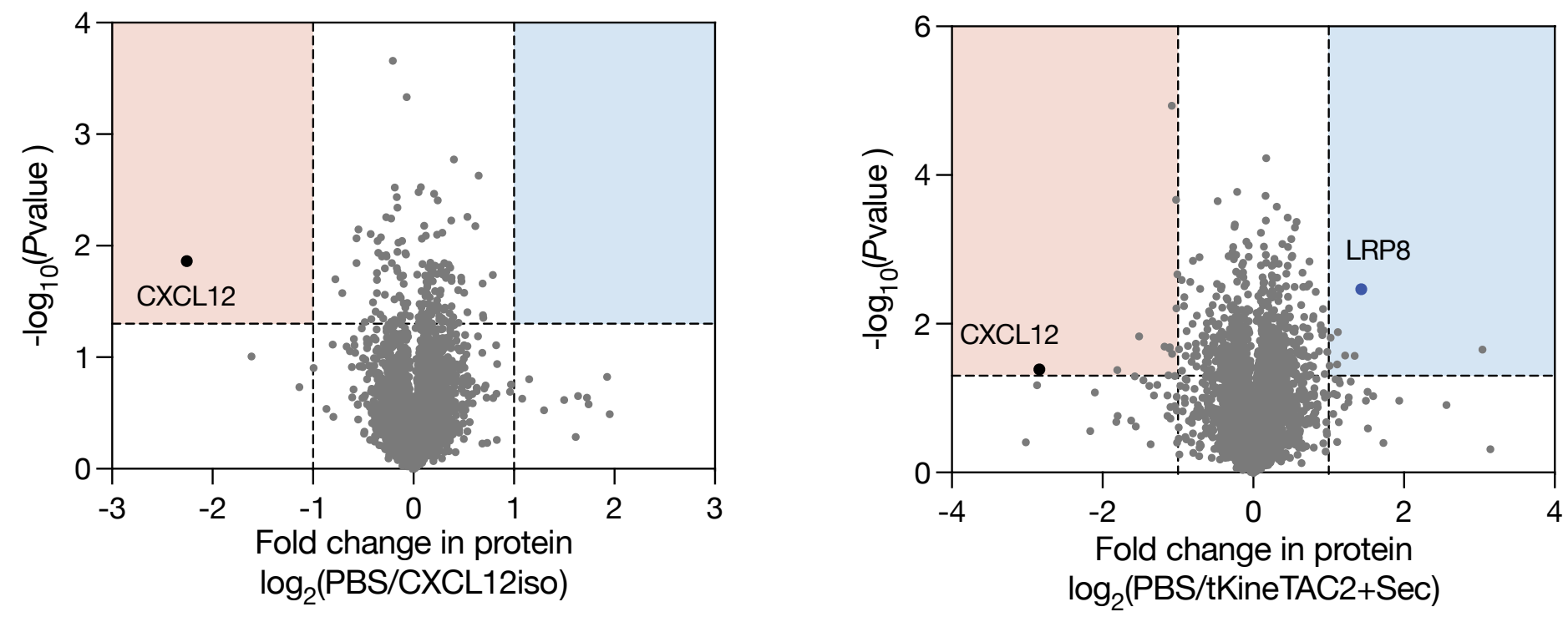

Fig. S7. Whole cell proteomics analysis of Kelly cells following KineTAC treatment. Kelly cells were treated for 48 hours with (left) 50 nM CXCL12 single-arm isotype antibody, or (right) 50 nM tKineTAC2 with 50 nM sodium selenite. Protein fold changes were quantified by mass spectrometry. Data are the mean of three biological replicates. P values were calculated using two-tailed Student's t-tests.

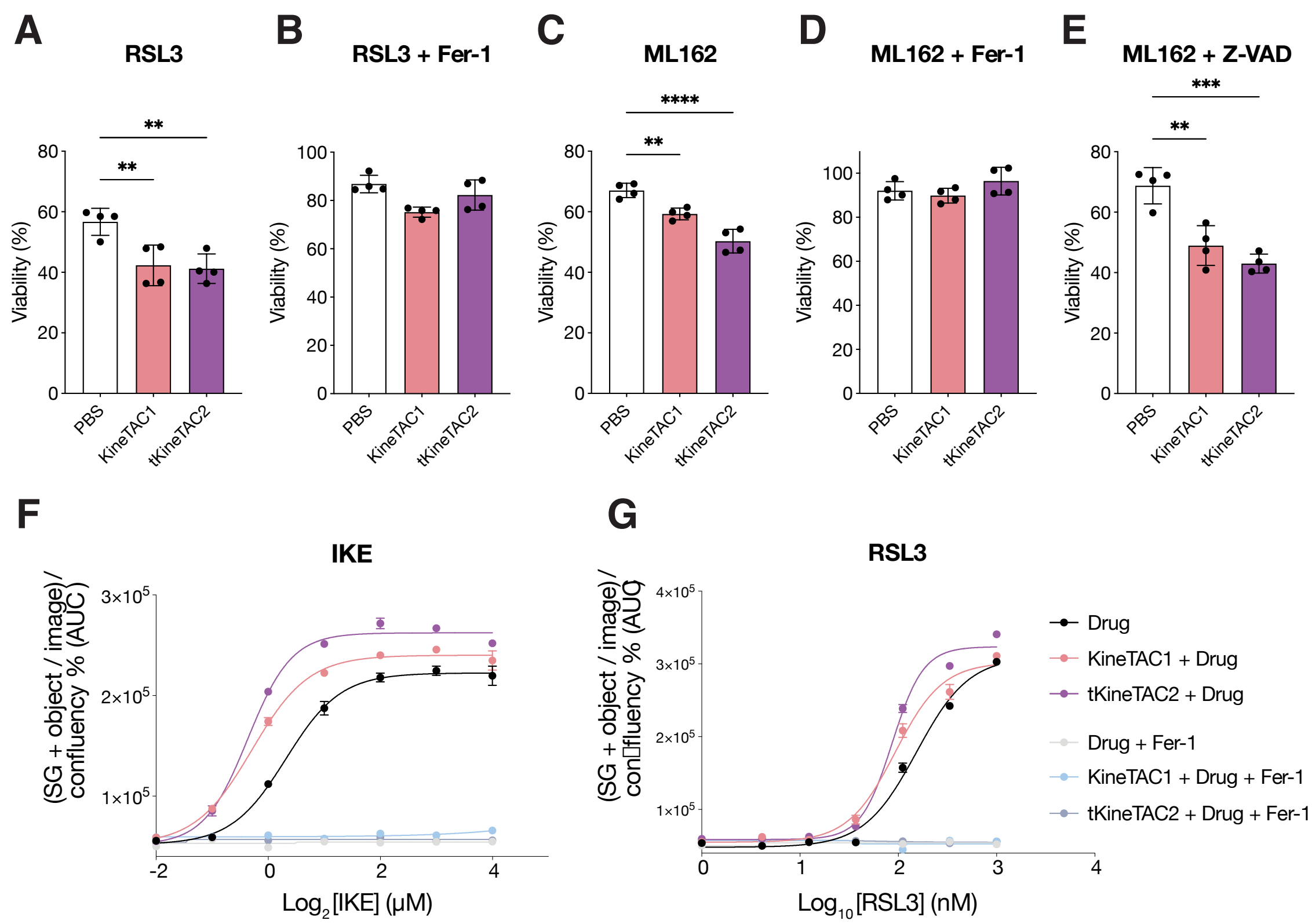

Fig. S8. Targeting LRP8 increases tumor cell vulnerability to ferroptosis. (A-E) Viability of HepG2 liver cells treated with KineTACs for 24 h, followed by 24 h treatment with 1 μM RSL3 **(A)**, 1 μM RSL3 + 1 μM Fer-1 **(B)**, 1 μM ML162 **(C)**, 1 μM ML162 + 1 μM Fer-1 **(D)**, or 1 μM ML162 + 5 μM Z-VAD-FMK respectively. Cell viability was measured using the CellTiter-Glo Reagent. Data represent quadruplicates ± SD. **(C)** Flow cytometry analysis showing that KineTACs were sufficient to increase lipid peroxidation of Kelly cells without ferroptosis inducers. Cells were treated with PBS or 50 nM KineTACs for 72 h, followed by 5 μM BODIPY 581/591 C11 staining. Statistics were determined by one-way ANOVA. **P < 0.01. ****P < 0.0001. **(C-D)** Incucyte-based analysis of Kelly cells viability following 72 h treatment with KineTAC1 or tKineTAC2, in combination with IKE **(C)** or RSL3 **(D)**, with or without Fer-1. Dead cells were labeled with the SYTOX green fluorescent dye. Data represent triplicate samples from three independent experiments.

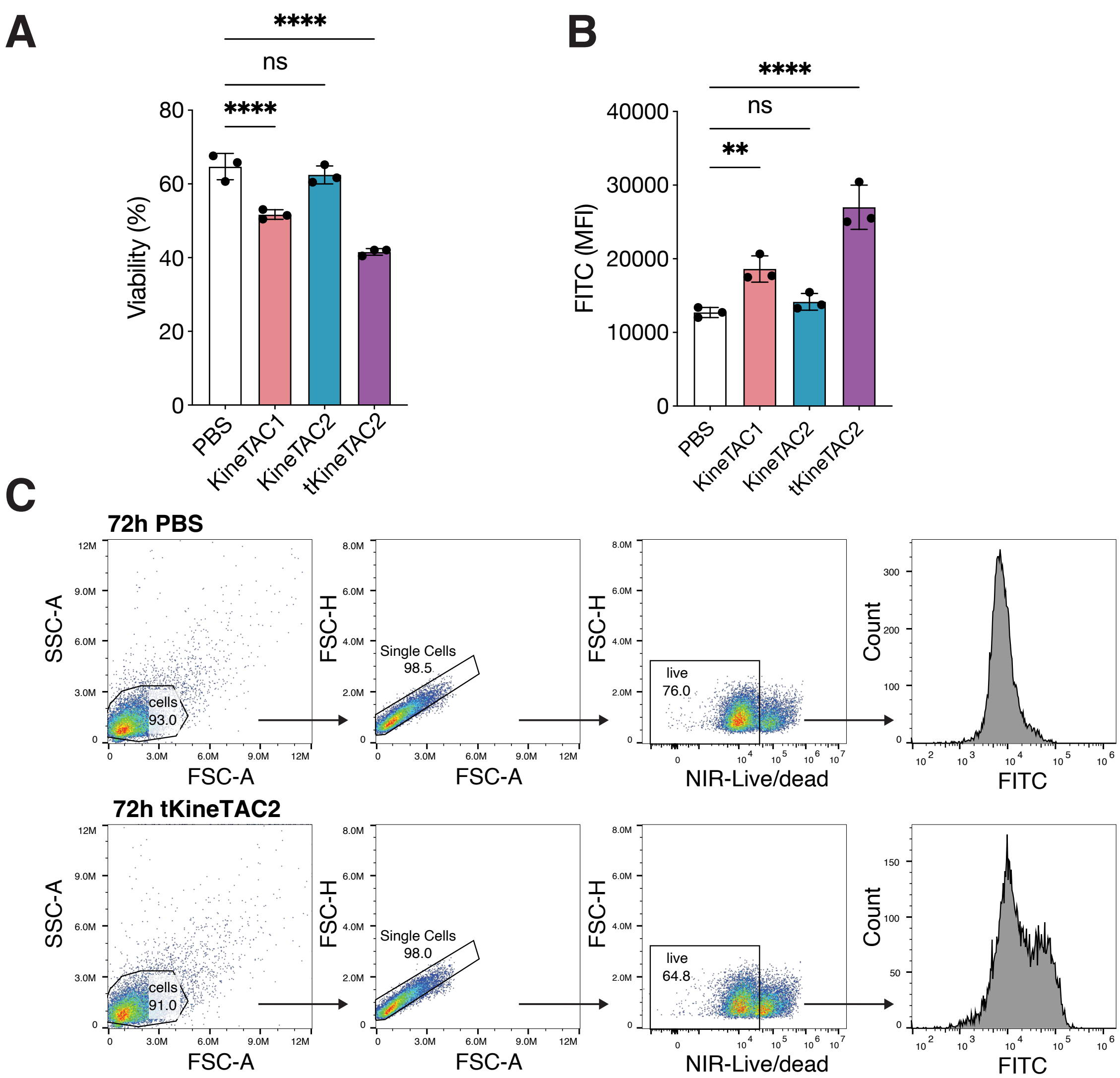

Fig. S9. Targeting LRP8 increases tumor cell vulnerability to ferroptosis. (A) Flow cytometry analysis of Kelly cell viability following KineTAC and RSL3 treatment, followed by Near-IR live/dead dye and BODIPY 581/591 C11 staining. **(B-C)** Flow cytometry analysis showing that KineTACs were sufficient to increase lipid peroxidation of Kelly cells without ferroptosis inducers. Cells were treated with PBS or 50 nM KineTACs for 72 h, followed by **(B)** 5 μM BODIPY 581/591 C11 staining. **(C)** Gating strategy of treated Kelly cells. Data represent mean ± SD of biological triplicates. Statistics were determined by one-way ANOVA. **P < 0.01. ****P < 0.0001. ns, not significant.

SI References

1. Y. Xu, *et al.*, Addressing polyspecificity of antibodies selected from an in vitro yeast presentation system: a FACS-based, high-throughput selection and analytical tool. *Protein Eng., Des. Sel.* **26**, 663–670 (2013).

2. P. M. Ridker, *et al.*, Lipid-Reduction Variability and Antidrug-Antibody Formation with Bococizumab. *N. Engl. J. Med.* **376**, 1517–1526 (2017).

3. T. F. Rogers, *et al.*, Isolation of potent SARS-CoV-2 neutralizing antibodies and protection from disease in a small animal model. *Science* **369**, 956–963 (2020).

4. Z. Li, *et al.*, Ribosome stalling during selenoprotein translation exposes a ferroptosis vulnerability. *Nat. Chem. Biol.* **18**, 751–761 (2022).
